# DNA Topoisomerase 1α Promotes Transcriptional Silencing of Transposable Elements through DNA Methylation and Histone Lysine 9 Dimethylation in *Arabidopsis*

**DOI:** 10.1371/journal.pgen.1004446

**Published:** 2014-07-03

**Authors:** Thanh Theresa Dinh, Lei Gao, Xigang Liu, Dongming Li, Shengben Li, Yuanyuan Zhao, Michael O'Leary, Brandon Le, Robert J. Schmitz, Pablo Manavella, Shaofang Li, Detlef Weigel, Olga Pontes, Joseph R. Ecker, Xuemei Chen

**Affiliations:** 1 Department of Botany and Plant Sciences, Center for Plant Cell Biology, Institute of Integrative Genome Biology, University of California Riverside, Riverside, California, United States of America; 2 ChemGen IGERT program, Center for Plant Cell Biology, Institute of Integrative Genome Biology, University of California Riverside, Riverside, California, United States of America; 3 School of Life Sciences, Lanzhou University, Lanzhou, China; 4 Plant Biology Laboratory, The Salk Institute for Biological Studies, La Jolla, California, United States of America; 5 Department of Molecular Biology, Max Planck Institute for Developmental Biology, Tübingen, Germany; 6 Department of Biology, University of New Mexico, Albuquerque, New Mexico, United States of America; 7 Howard Hughes Medical Institute, The Salk Institute for Biological Studies, La Jolla, California, United States of America; 8 Howard Hughes Medical Institute, University of California Riverside, Riverside, California, United States of America; ETHZ, Switzerland

## Abstract

RNA-directed DNA methylation (RdDM) and histone H3 lysine 9 dimethylation (H3K9me2) are related transcriptional silencing mechanisms that target transposable elements (TEs) and repeats to maintain genome stability in plants. RdDM is mediated by small and long noncoding RNAs produced by the plant-specific RNA polymerases Pol IV and Pol V, respectively. Through a chemical genetics screen with a luciferase-based DNA methylation reporter, *LUCL*, we found that camptothecin, a compound with anti-cancer properties that targets DNA topoisomerase 1α (TOP1α) was able to de-repress *LUCL* by reducing its DNA methylation and H3K9me2 levels. Further studies with *Arabidopsis top1α* mutants showed that TOP1α silences endogenous RdDM loci by facilitating the production of Pol V-dependent long non-coding RNAs, AGONAUTE4 recruitment and H3K9me2 deposition at TEs and repeats. This study assigned a new role in epigenetic silencing to an enzyme that affects DNA topology.

## Author Summary

DNA topoisomerase is an enzyme that releases the torsional stress in DNA generated during DNA replication or transcription. Here, we uncovered an unexpected role of DNA topoisomerase 1α (TOP1α) in the maintenance of genome stability. Eukaryotic genomes are usually littered with transposable elements (TEs) and repeats, which pose threats to genome stability due to their tendency to move or recombine. Mechanisms are in place to silence these elements, such as RNA-directed DNA methylation (RdDM) and histone H3 lysine 9 dimethylation (H3K9me2) in plants. Two plant-specific RNA polymerases, Pol IV and Pol V, generate small and long noncoding RNAs, respectively, from TEs and repeats. These RNAs then recruit protein factors to deposit DNA methylation or H3K9me2 to silence the loci. In this study, we found that treatment of plants with camptothecin, a TOP1α inhibitor, or loss of function in *TOP1α*, led to the de-repression of RdDM target loci, which was accompanied by loss of H3K9me2 or DNA methylation. The role of *TOP1α* in RdDM could be attributed to its promotion of Pol V, but not Pol IV, transcription to generate long noncoding RNAs.

## Introduction

DNA methylation and histone H3 lysine 9 (H3K9) methylation are two chromatin modifications widely employed by eukaryotes to maintain genome stability [1], [2]. H3K9 methylation and DNA methylation are targeted via small interfering RNAs (siRNAs) to repeats and transposable elements (TEs) and are required for their transcriptional silencing [1], [2].

In plants, cytosine methylation is established through a process known as RNA-directed DNA methylation (RdDM), which involves small and long noncoding RNAs produced by plant-specific RNA polymerases, Pol IV and Pol V, respectively [2]. Pol IV is thought to transcribe RdDM target loci and generate long precursor RNAs. These are eventually processed into 24-nucleotide (nt) siRNAs that are loaded into the Argonaute protein AGO4 [3], [4], [5], [6], [7]. In parallel, Pol V generates long non-coding RNA transcripts from RdDM target loci, and these transcripts recruit siRNA-AGO4 to chromatin [8], [9]. Through the concerted action of these two polymerases, siRNA-AGO4 becomes localized to target loci, and this ultimately recruits the methyltransferase DRM2, which effects *de novo* DNA methylation. In plants, DNA methylation occurs in three sequence contexts, CG, CHG, and CHH. In contrast to CG and CHG methylation, which can be maintained through the DNA methyltransferases MET1 and CMT3, respectively, CHH methylation is propagated by constant *de novo* methylation through RdDM [2], [10].

In plants, H3K9 dimethylation (H3K9me2) is another repressive chromatin mark associated with TE and repeat silencing [11], [12], [13]. H3K9me2 and CHG methylation act in a self-reinforcing loop to promote the maintenance of these marks by histone methyltransferases KRYPTONITE (KYP or SUVH4), SUVH5 and SUVH6 and the DNA methyltransferase CMT3 [14]. How H3K9me2 is initially deposited is less well understood, but the RdDM pathway plays a role, as mutations in RdDM pathway genes cause marked reductions in H3K9me2 levels at RdDM target loci [7], [8], [15]. In fact, a recent study revealed a strong genome-wide inter-dependence between non-CG (CHG and CHH) DNA methylation and H3K9 dimethylation [16].

DNA topoisomerases are enzymes that maintain proper DNA topology [17]. During replication or transcription, the DNA helical structure opens to form the replication or transcription fork, and the DNA in front of the fork becomes positively supercoiled, while the DNA behind the fork becomes negatively supercoiled. Topoisomerases bind these regions, nick the DNA to relieve the torsional stress, and re-ligate the DNA. Topoisomerases are divided into two major types, I and II, and further subtypes depending on their mode of action and structure [17], [18].

In *Arabidopsis*, there are two genes encoding type IB topoisomerases, *TOP1α* and *TOP1β*, which are tandemly arrayed in the genome. *top1α* mutants exhibit gross morphological defects, while *top1β* mutants are phenotypically normal [19]. RNAi-mediated knockdown of *TOP1β* in a *top1α* background is lethal [19]; thus these two genes are functionally redundant.

Here, we uncover a role of *TOP1α* in transcriptional silencing of TEs. We exploited a luciferase-based reporter (*LUCL*) that undergoes transcriptional silencing by DNA methylation [20] to perform a chemical genetics screen. We found that camptothecin (CPT) released the DNA methylation of *LUCL* and de-repressed its expression. CPT is a well-studied natural quinoline alkaloid that targets type 1B topoisomerases [21], [22]. Both the addition of CPT and loss-of-function in *TOP1α* led to the de-repression of RdDM target loci accompanied by a release of DNA methylation and/or a decrease in H3K9me2 levels. *TOP1α* is dispensable for Pol IV-mediated siRNA biogenesis but is required for the production of Pol V-dependent, long non-coding RNA transcripts. Consistent with the current model that these transcripts recruit siRNA-AGO4 to chromatin, inactivation of *TOP1α* resulted in reduced AGO4 occupancy at these loci. Taken together, through the identification of *TOP1α* as a player in RdDM, we have assigned new roles to a protein affecting DNA topology.

## Results

### Camptothecin releases the silencing of *LUCL*

To identify genes involved in DNA methylation, we performed a chemical genetics screen with *LUCL*, a transcriptionally-silenced luciferase (*LUC*)-based reporter line [20]. In *LUCL*, *LUC* is driven by a dual *35S* promoter and both the *35S* promoter and the *LUC* coding region harbor DNA methylation [20]. The DNA methylation at *LUCL*, and consequently its transcriptional silencing, is controlled by *MET1*, and to a lesser extent, by the RdDM pathway [20].

**Figure 1:**
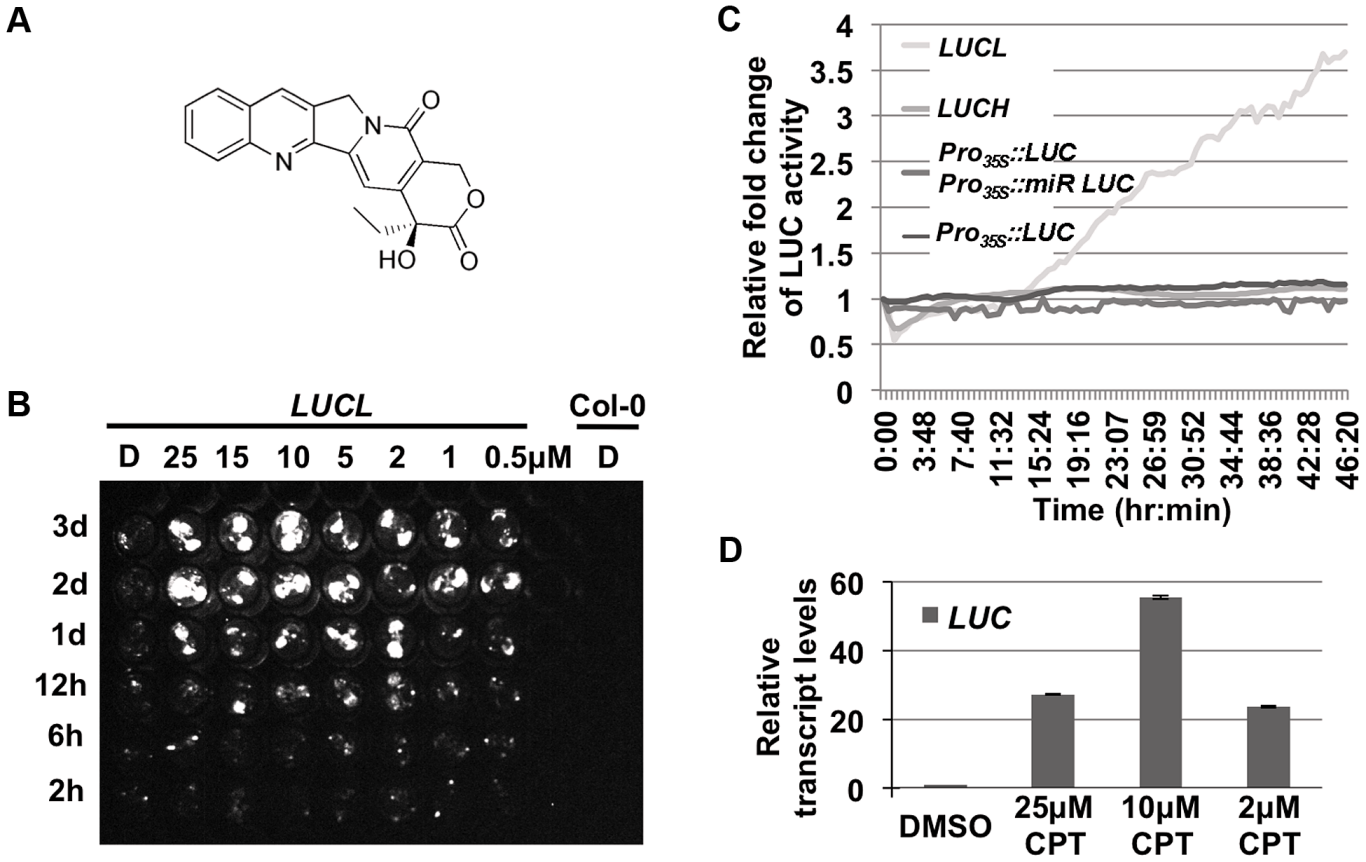
CPT releases LUC activity of *LUCL*. (A) Chemical structure of CPT. (B) Time- and concentration-dependent de-repression of *LUCL* by CPT. Seedlings were subjected to CPT treatment in 96-well plates and imaged for LUC activity at different time points (shown to the left of the image) after CPT addition. D = DMSO-treated seedlings. Col-0 wild type (without the *LUCL* transgene) was included as a negative control. (C) Effects of CPT treatment on various transgenes. LUC activity was measured over time using an automated luciferase reader. The x-axis indicates the time of incubation in terms of hours:minutes after addition of 10 µM CPT or DMSO. The y-axis indicates the relative fold change of LUC activity upon treatment of CPT as compared to DMSO. CPT affects the activity of *LUCL* but not *Pro_35S_:LUC Pro_35S_:mir-LUC*, a reporter line in which the *LUC* transgene reports miRNA activity [23], *Pro_35S_:LUC*, [23], or *LUCH*, a reporter line in which the *LUC* transgene is silenced specifically by CHH methylation [36]. (D) qRT-PCR measuring *LUC* transcript levels upon addition of different concentrations of CPT. Three biological replicates, each with three technical replicates, were performed. Error bars represent standard deviation from the three biological replicates.

Over 3,000 compounds were screened against *LUCL* seedlings for their effects on *LUC* expression. A hit compound, camptothecin (CPT) (Figure 1A), was found to release *LUC* silencing in a concentration- and time-dependent manner (Figure 1B and C). Interestingly, CPT released *LUC* silencing in a bi-phasic manner, with optimal levels at 10 µM. Further, the release of LUC activity was not observed until one day of chemical addition in a time course assay (Figure 1B). Consistently, continuous live imaging revealed that an increase in LUC activity occurred at about 15 hr after the addition of the chemical (Figure 1C). The slow kinetics suggested that cell division is likely necessary for the de-repression of the reporter. The effects of CPT on LUC protein activity reflected a release of *LUCL* silencing, as the addition of CPT led to an increase in *LUC* transcript levels (Figure 1D). Consistent with the dose-dependent effects of CPT on LUC activity, *LUC* transcript levels were most de-repressed at 10 µM of CPT (Figure 1D).

Previous experiments with *LUCL* ruled out that it reports miRNA activity, even though it contains the miR172 binding sequence [20]. Consistently, we found that the addition of CPT did not release the LUC activity of a miRNA reporter line, *Pro_35S_::LUC Pro_35S_::miR-LUC* (Figure 1C; [23]). Thus, CPT released the LUC activity of *LUCL* through a miRNA-independent mechanism.

**Figure 2:**
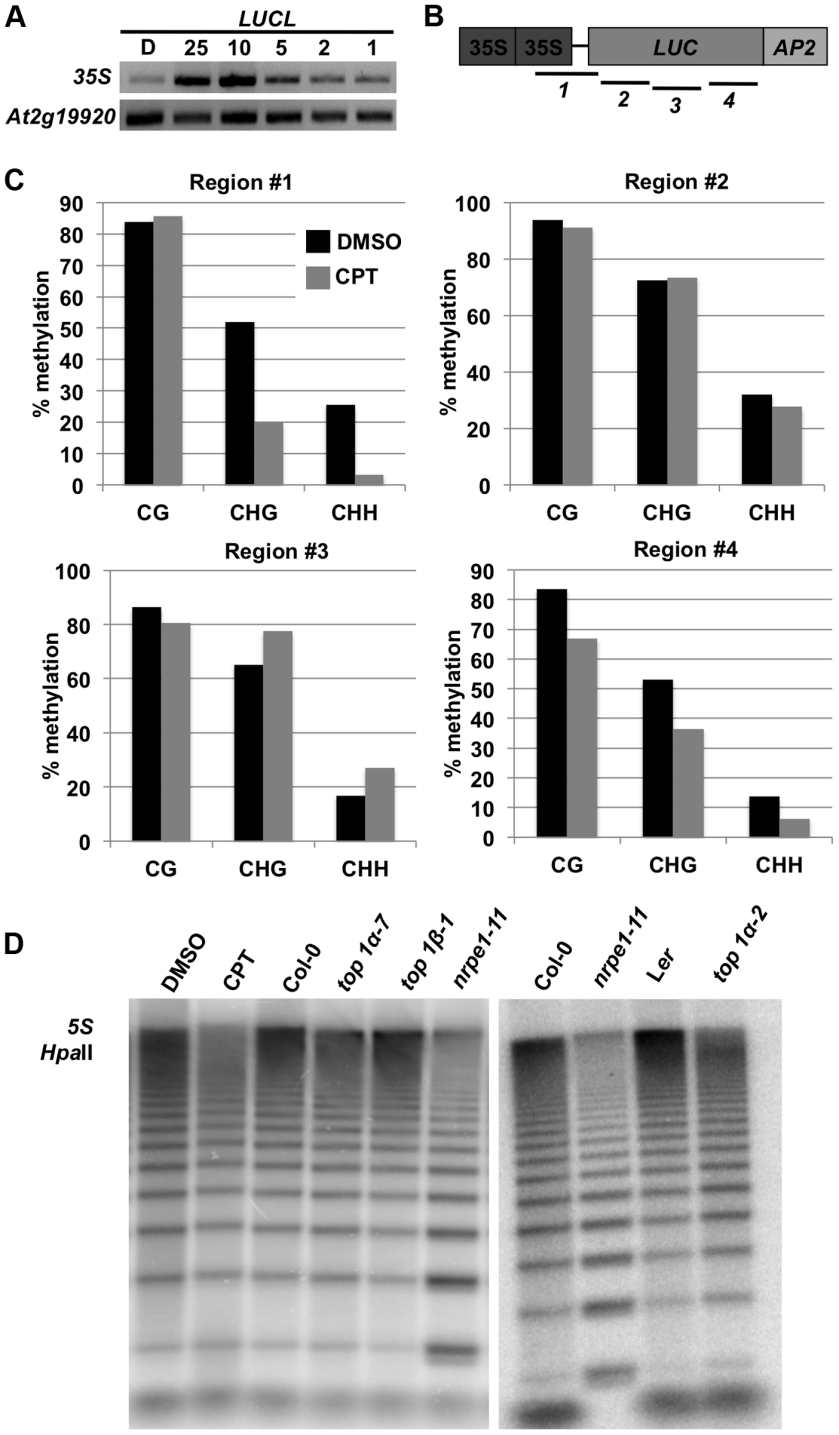
Addition of CPT or loss of *TOP1α* releases DNA methylation. (A) McrBC-PCR-based methylation analysis of the *35S* promoter in *LUCL* seedlings treated with CPT. *At2g19920* is unmethylated and serves as an internal loading control. The number listed above indicates the concentration (in µM) of CPT added. D = DMSO. (B) A schematic diagram of the *LUCL* transgene. The numbered black lines indicate the regions for which bisulfite sequencing was performed (Figure 2C). (C) Levels of DNA methylation of *LUCL* in DMSO- and CPT-treated seedlings as determined by bisulfite sequencing. The regions correspond to the numbered lines in Figure 2B. (D) Loss of *TOP1α* results in reduced *5S* rDNA methylation. Genomic DNA was digested with *Hpa*II followed by Southern blotting. Less methylated DNA is expected to yield a higher intensity of bands lower down the gel as in *nrpe1-11* (a Pol V mutant). In the CPT-treated sample, 25 µM of CPT was used. *top1α-7*, *top1β-1*, and *nrpe1-11* are to be compared to Col-0 (wild type), and *top1α-2* is to be compared to L*er* (wild type).

To determine whether CPT increased *LUC* transcript levels by reducing DNA methylation, we performed McrBC-PCR to examine the methylation status of *LUCL*. After digestion of genomic DNA with McrBC, an enzyme that only cuts methylated DNA [24], *35S* promoter sequences were amplified by PCR. In the DMSO-treated control sample, little product was observed, indicating that this region was highly methylated in *LUCL*. However, after CPT treatment, the amount of PCR products increased (Figure 2A), suggesting that CPT treatment led to a reduction in *35S* promoter methylation. In addition, the DNA methylation status of the *35S* promoter and the *LUC* coding region was examined by bisulfite sequencing (Figure 2B). The addition of 10 µM CPT resulted in a drastic reduction of CHH methylation, and to some extent CHG methylation, in region #1 (Figure 2C). CG methylation was largely unaffected upon CPT treatment, with the exception of region #4 (Figure 2C).

### The CPT target, TOP1α, promotes CG methylation at *5S* repeats

Due to their potent anti-cancer properties, CPT and its analogs have been intensely studied. The cellular target of CPT is topoisomerase I and the mechanism by which CPT inhibits topoisomerase I is well understood [25]. Given this knowledge, our finding that CPT de-represses *LUCL* implicated *TOP1α* in transcriptional gene silencing. A *top1α* mutant allele, *top1α-2*, had been found in an unrelated project (Xigang Liu and Xuemei Chen, unpublished results). The *top1α-2* mutant carried a C→T point mutation in the second exon, which generates a premature stop codon (Figure S1A). *top1α-2*, which had been isolated in the Landsberg *erecta* background, was introgressed into Col-0 through five backcrosses to derive *top1α-2^Col^*. *top1α-2^Col^* was then crossed to *LUCL* in the Col-0 background. Unlike CPT, which released LUC activity, the *top1α-2^Col^* mutation was not able to release LUC activity (Figure S1B), probably due to activity of the partially redundant *TOP1β* gene.

We next asked whether TOP1α inactivation or CPT treatment affected DNA methylation of endogenous RdDM loci. *5S* rDNA is present with thousands of copies in the genome and is under RdDM regulation [5]. We digested genomic DNA with *Hpa*II, an enzyme that cuts unmethylated DNA in a CG context, to determine the status of *5S* rDNA methylation. We found that, like *nrpe1-11*, a Pol V mutant, *top1α-2* and CPT-treated seedlings had less methylated DNA, as indicated by the increase in intensity of the lower molecular weight restriction fragments (Figure 2D). *top1α-7* (also known as *mgo1-7* [26]; Figure S1A) has weaker developmental defects than *top1α-2^Col^*. The *top1β-1* loss-of-function mutant in the Col-0 background (Figure S1A) has no obvious morphological defects (Xigang Liu and Xuemei Chen, unpublished results). CG methylation at *5S* repeats was only weakly reduced in *top1α-7* mutants and unaffected in *top1β-1* mutants. Similarly, DNA blot analyses were conducted to examine CHG methylation at *MEA-ISR* and *180 bp* repeats (Figure S1C and D), and CHH methylation at 5S rDNA repeats. Only a slight reduction in CHG methylation at the *180 bp* repeats was detected in *top1α-2* (Figure S1D). *top1α-2* was indistinguishable from the isogenic L*er* parental line in terms of CHG methylation at *MEA-ISR* (Figure S1C) or CHH methylation at *5S* repeats (Figure S1D).

### TOP1α has a limited role in DNA methylation in the genome

The studies above on a small number of loci revealed a limited role of *TOP1α* in DNA methylation. In order to obtain a global view of the function of *TOP1α* in DNA methylation, we performed whole genome bisulfite sequencing (MethylC-seq) on L*er*, *top1α-2*, Col-0, *top1α-7*, *nrpd1-3* (a Pol IV mutant) and *nrpe1-11* seedlings. A total of 10 libraries representing one to three biological replicates of the genotypes (Table S1) were sequenced. Acceptable bisulfite conversion efficiency (Table S1) and read coverage (Table S2) were achieved for each library.

**Figure 3:**
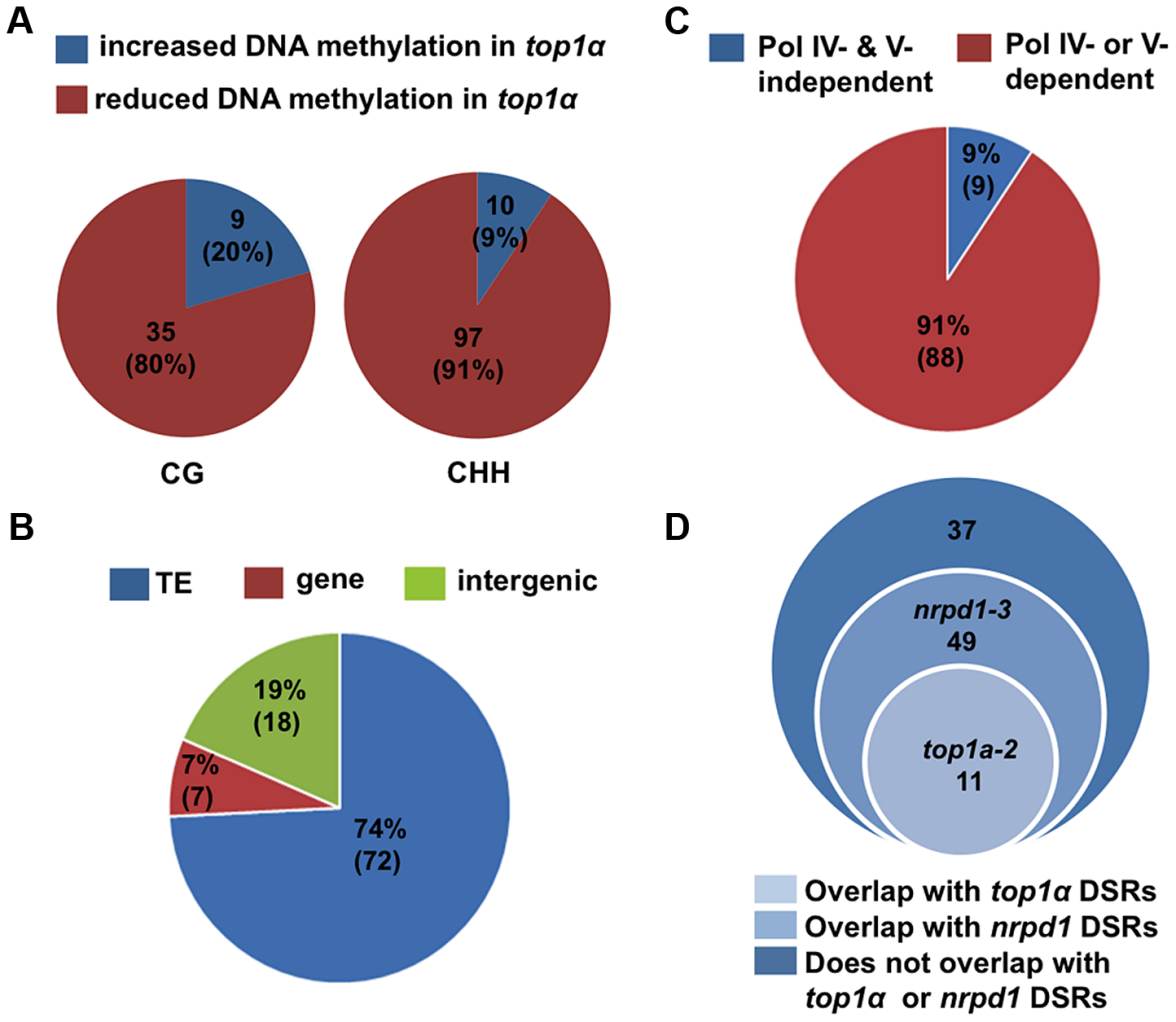
*TOP1α* does not globally impact DNA methylation but promotes CHH methylation at a small number of loci. (A) Pie charts showing that the great majority of WT- *top1α* DMRs show reduced DNA methylation in *top1α*. Each circle represents total WT- *top1α* DMRs in a methylation context (CG or CHH). The red and blue areas represent DMRs with reduced and increased DNA methylation in *top1α*, respectively. The numbers indicate the numbers of DMRs in each category. The numbers in the parentheses represent the percentage of the DMR category in total DMRs. (B) The majority of WT- *top1α* CHH DMRs showing reduced DNA methylation in *top1α* overlap with TEs (74%, blue). Those that overlap with genes and intergenic regions are shown in red (7%) and green (19%), respectively. (C) The majority of WT- *top1α* CHH DMRs overlap with CHH DMRs between WT and *nrpd1* or WT and *nrpe1* (91%, red), suggesting that these regions require Pol IV or Pol V for CHH methylation. The portion of WT- *top1α* CHH DMRs not overlapping with WT-*nrpd1* or WT-*nrpe1* CHH DMRs are shown in blue (9%). (D) Overlap between WT- *top1α* CHH DMRs with DSRs (differential small RNA regions) between WT and *nrpd1* or WT and *top1α*. There is little overlap between WT-*top1α* CHH DMRs and WT-*top1α* DSRs.

We identified differentially methylated regions (DMRs) using established procedures in the literature (see Material and Methods and Text S1). We compared each mutant to its wild-type control in the same biological replicate. We also called DMRs among the three Col-0 replicates to establish the background of spontaneous DMRs in wild type. Despite the high degree of reproducibility of the biological replicates (Table S3), when the three Col-0 replicates were subjected to the DMR analysis, we found thousands of CHH DMRs, but very few CG and CHG DMRs, between any two Col-0 replicates (Table S4A). In MethylC-seq data of three Col-0 replicates from a published study [27], we also identified thousands of CHH DMRs between any two replicates (Table S4B). This suggested that CHH methylation is considerably variable. In light of such variability, we took a conservative approach towards the identification of robust DMRs by considering only the overlap between two biological replicates or mutant alleles. For example, to derive DMRs between wild type and *nrpd1-3* or *nrpe1-11*, we first compared the mutant to wild type within each biological replicate and then retained only DMRs that overlapped in both biological replicates (Table S5B and C). To derive DMRs between wild type and *top1α*, we first compared *top1α-7* to Col-0 and *top1α-2* to L*er*, and then obtained the overlapped DMRs between the two alleles (Table S5A). In addition, hypervariability (HV) regions that are prone to changes in DNA methylation over generations [28], [29] were subtracted from the overlapped DMRs. The final set of CHH DMRs between wild type and *nrpd1-3* (or *nrpe1-11*) consisted of over 7,500 loci showing reduced DNA methylation in the mutants (Table S5B and C), consistent with the known roles of Pol IV and Pol V in CHH methylation [27], [30]. The final set of DMRs between wild type and *top1α* consisted of the following: reduced in methylation in *top1α* — 97 (CHH), 35 (CG), and 0 (CHG); and increased in methylation in *top1α* — 10 (CHH), 9 (CG), and 1 (CHG) (Figure 3A, Table S5A and Table S6). The overall change in CHH methylation in *top1α* was very limited in comparison to that in *nrpd1-3* or *nrpe1-11* (Table S5). Most of the 97 WT-*top1α* CHH DMRs are in TEs or intergenic regions (Figure 3B). 91% of the WT-*top1α* CHH DMRs require Pol IV or Pol V for their CHH methylation (Figure 3C). This suggested that TOP1α promotes DNA methylation at a small number of RdDM loci.

### TOP1α silences transposons through DNA methylation and H3K9 dimethylation

**Figure 4:**
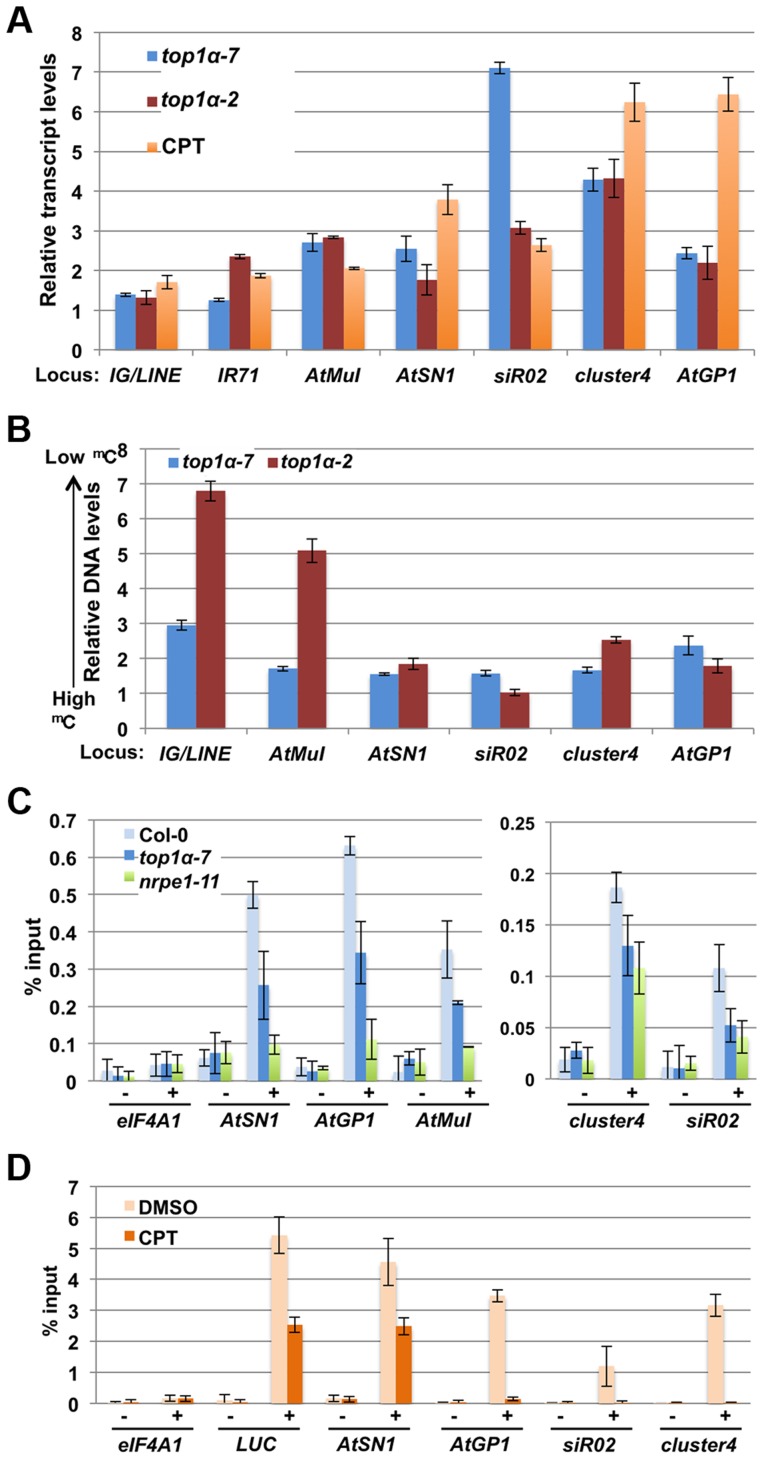
TOP1α promotes transposon silencing at endogenous RdDM target loci through H3K9me2 deposition. In (A) to (D), error bars represent standard deviation calculated from three biological replicates, each with three technical replicates. In (A) and (B), *top1α-7*, *top1α-2* and CPT-treated *LUCL* were compared to Col-0, L*er*, and DMSO-treated *LUCL* respectively. The relative levels to these controls (set to 1.0) are shown. The loci tested are labeled on the x axis. (A) Loss of *TOP1α* or addition of CPT results in RdDM target loci de-repression. (B) Loss of *TOP1α* or addition of CPT results in a release of DNA methylation at some loci. McrBC-qPCR analysis was performed to quantify DNA methylation levels in *top1α* or CPT-treated plants. Higher DNA levels in this assay correlate with lower levels of DNA methylation. *At1g40129* served as an internal unmethylated control. (C) Loss of *TOP1α* results in reduced H3K9me2 levels at endogenous RdDM loci. ChIP-qPCR was performed to measure H3K9me2 levels at five RdDM target loci. *eIF4A1*, which does not harbor H3K9me2, was used as an internal control. − = samples processed without antibody. + = samples processed with anti-H3K9me2 antibodies. (D) CPT treatment results in reduced H3K9me2 levels at the *LUCL* transgene and four endogenous RdDM loci. *LUCL* plants were treated with either DMSO or 10 µM CPT and subjected to ChIP. qPCR was performed with the immunoprecipitated DNA for the *LUCL* transgene and four endogenous RdDM loci. *eIF4A1* was used as an internal negative control. − = samples processed without antibody. + = samples processed with anti-H3K9me2 antibodies.

Since the methylation-sensitive DNA blot analyses only revealed an effect of *top1α* alleles on DNA methylation at the *5S* and *180 bp* repeats and the methylome profiling studies did not support a global role of TOP1α in DNA methylation, we sought to evaluate whether TOP1α is required for the transcriptional silencing of endogenous RdDM loci. qRT-PCR was performed to determine transcript levels from seven well-known RdDM loci. In both wild-type seedlings treated with CPT as well as *top1α* (both *top1α-2* and *top1α-7*) seedlings, these endogenous siRNA target loci were de-repressed (Figure 4A). This confirmed a role of TOP1α in silencing the RdDM target loci.

We asked whether the release of transcriptional silencing of endogenous RdDM target loci (Figure 4A) in *top1α* or CPT-treated seedlings was accompanied by a loss of DNA methylation. We performed McrBC-qPCR assays to quantify the levels of DNA methylation amongst different genotypes/treatments at six endogenous RdDM loci. At most of the loci, DNA methylation was reduced in the two *top1α* mutants, but the reductions were small in *top1α-7* (Figure 4B). Treatment of wild-type (L*er*) plants with CPT resulted in reductions in DNA methylation at four of the six tested loci (Figure S1E). Although the overall trend of reduced DNA methylation in the two *top1α* mutants and CPT treated plants agreed with the observed de-repression of these loci, there were also inconsistencies whereby de-repression was not accompanied by reductions in DNA methylation, such as at siR02 in *top1α-2* and CPT-treated plants.

This incomplete correlation between TE de-repression and a reduction in DNA methylation prompted us to ask whether *TOP1α* silences TEs through another mechanism. Previous studies have shown that H3K9me2 is a major repressive mark for transposon silencing and that H3K9me2-dependent silencing acts in concert or in parallel with RdDM [31], [32], [33]. Like DNA methylation, H3K9me2 is targeted to specific TEs through siRNA-AGO4 [7]. Thus, we investigated whether loss of *TOP1α* function or CPT treatment altered H3K9me2 levels at TEs. Chromatin immunoprecipitation (ChIP)-qPCR showed that H3K9me2 levels at *AtSN1*, *sir02*, cluster4, *AtGP1*, and *AtMuI* were reduced in both *top1α-7* and *nrpe1-11* (Figure 4C). We also performed ChIP-qPCR on *LUCL* seedlings treated with DMSO or CPT. CPT treatment was found to cause a strong reduction in H3K9me2 levels at four TE loci (Figure 4D). As CPT was initially isolated through a chemical genetics screen with *LUCL*, we asked whether the *LUC* transgene in *LUCL* also harbored H3K9me2 and, if so, whether CPT treatment reduced its H3K9me2 levels. Indeed, ChIP-qPCR showed that the *d35S* of the *LUC* transgene (region #1 in Figure 2B) harbored H3K9me2, with CPT treatment reducing H3K9me2 levels (Figure 4D).

As H3K9me2, which is introduced by KYP and its paralogs, and CHG methylation, which is deposited by CMT3, act in a self-reinforcing loop, and both H3K9me2 and CMT3 contribute to CHH methylation [14], [16], we asked whether the role of *TOP1α* in DNA methylation depends on KYP or CMT3. To address this question, we treated L*er* (wild-type), *kyp-2* and *cmt3-7* plants with CPT to inhibit topoisomerase I activity and then assayed DNA methylation at six TE loci. CPT treatment of wild-type plants resulted in reduced DNA methylation at four of the six loci (Figure S1E). The reduction in DNA methylation caused by CPT treatment was minimal at these four loci in either *cmt3-7* or *kyp-2* (Figure S1E). This suggested that the effects of *TOP1α* in DNA methylation require CMT3- and KYP-mediated H3K9 dimethylation.

### TOP1α does not affect small RNA levels

The promotion of DNA methylation and/or H3K9me2 deposition at TEs implicates a role of TOP1α in RdDM, a process that involves Pol IV and Pol V. As topoisomerases are required to release DNA topological tension generated by transcription [17], it would be reasonable to expect that TOP1α is required for the activities of either Pol IV or Pol V. We first tested whether TOP1α is required for the activities of Pol IV, the output of which is the accumulation of 24-nt siRNAs from RdDM target loci. RNA blot analysis showed that siRNA accumulation at several loci was similar in L*er* and *top1α-2* (Figure S3A). To gain a global view on the potential relationship between TOP1α and Pol IV, we compared deep sequencing profiles of small RNAs from L*er*, *top1α-2*, Col-0, *nrpd1-3*, and *nrpe1-11*. The size distributions of all small RNA reads in L*er* and *top1α-2* were almost identical (Figure S3B). To determine whether TOP1α affects siRNA accumulation at specific regions of the genome, we identified differential small RNA regions (DSRs). While large numbers of DSRs were found in *nrpd1-3* or *nrpe1-11* relative to the wild-type control, consistent with the essential role of Pol IV and the auxiliary role of Pol V in siRNA biogenesis [3], [5], [6], very few were found in *top1α-2* (Table S7). Furthermore, analysis of small RNA abundance throughout the genome did not support a global role of TOP1α in small RNA accumulation (Figure S3C). Therefore, Pol IV activity does not appear to require TOP1α.

Given that we had found 71 WT-*top1α* DSRs (Table S7), we asked whether the reduced CHH methylation at the 97 WT-*top1α* DMRs was associated with reduced siRNA levels. We found that only 11 of the 97 DMRs overlapped with WT-*top1α* DSRs (Figure 3D). A representative of such a locus is shown in Figure S2A. Most of the 97 DMRs did not overlap with the 71 WT- *top1α* DSRs; two such loci are shown in Figure S2B and C. Therefore, the reduced CHH methylation in *top1α* could not be explained by reduced siRNA levels. On the other hand, more than 60% of the 97 WT-*top1α* DMRs overlapped with WT-*nrpd1* DSRs (Figure 3D; Figure S2A and B), suggesting that these regions, which require TOP1α for CHH methylation, undergo Pol IV-dependent siRNA production. Therefore, TOP1α must promote CHH methylation at these RdDM loci independently of siRNA biogenesis.

### TOP1α promotes the production of Pol V-dependent transcripts and AGO4 occupancy at TEs

**Figure 5:**
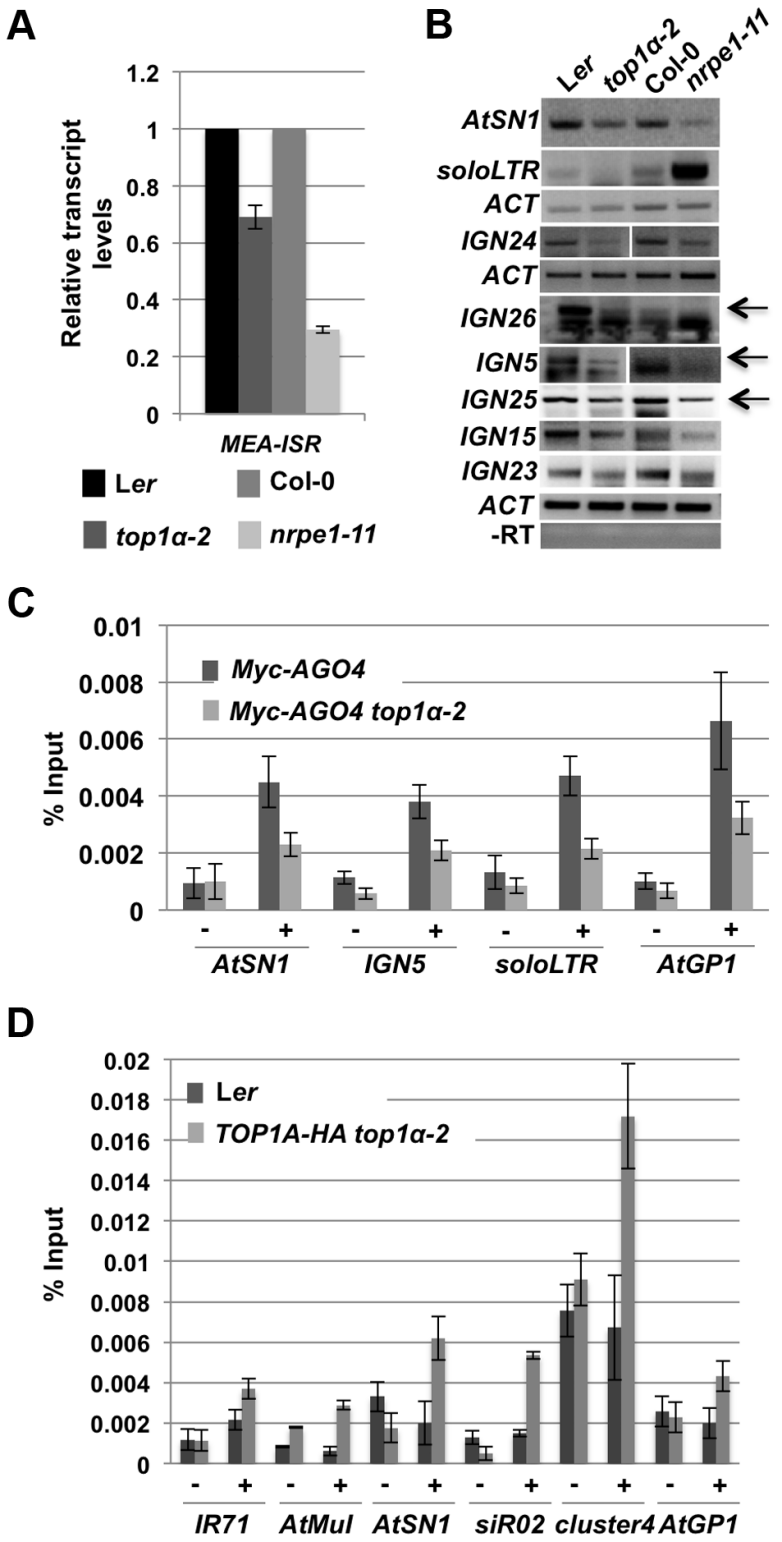
*TOP1α* promotes the production of Pol V-dependent transcripts and AGO4 occupancy at RdDM loci. (A) *TOP1α* contributes to the production of Pol V-dependent transcripts at *MEA-ISR*. qRT-PCR was performed to quantify Pol V-dependent transcripts at *MEA-ISR*. The Pol V mutant *nrpe1-11* was included as a control. *nrpe1-11* and *top1α-2* should be compared to the wild-type strains Col-0 and L*er*, respectively. Error bars represent standard deviation calculated from three technical replicates. (B) *TOP1α* contributes to the production of Pol II- or Pol V-dependent transcripts at many loci (noted on the left of the gel images). RT-PCR was performed to detect Pol II-dependent transcripts at *soloLTR* and Pol V-dependent transcripts at the other seven loci (*AtSN1* and six *IGN* loci). When multiple bands are present, the band representing the Pol V-dependent transcript is indicated by an arrow. Actin (*ACT*) served as an internal loading control for all the gels above. *nrpe1-11* was included as a control. *nrpe1-11* and *top1α-2* should be compared to the wild-type strains Col-0 and L*er*, respectively. –RT is the negative control in which reverse transcription was conducted in the absence of reverse transcriptase. Three biological replicates were performed and one representative image is shown. (C) Loss of *TOP1α* results in a decrease in AGO4 occupancy. Ten-day old wild-type and *top1α-2* seedlings, both of which contain a *Myc-AGO4* transgene, were subjected to ChIP with anti-MYC antibodies. qPCR was then performed on the immunoprecipitated DNA for four endogenous RdDM loci. “−” and “+” signs represent “no antibody” or “anti-Myc antibodies”, respectively. Error bars represent standard deviation calculated from three biological replicates. (D) TOP1α is present at six RdDM loci. Ten-day old L*er* (used as a negative control) and *TOP1A-HA top1α-2* seedlings were subjected to ChIP with anti-HA antibodies. qPCR was then performed on the immunoprecipitated DNA for six endogenous RdDM loci. “−” and “+” signs represent “no antibody” or “anti-HA antibodies”, respectively. Error bars represent standard deviation calculated from three technical replicates. Two biological replicates were performed and showed the same trend. One biological replicate is shown.

We next tested whether TOP1α promotes the production of Pol V-dependent transcripts. We performed qRT-PCR and RT-PCR to detect Pol V-dependent transcripts from eight loci, *MEA-ISR*, *AtSN1*, and six *IGN* loci that produce such transcripts [9], [30]. At all eight loci, the levels of the Pol V-dependent transcripts were reduced in *top1α-2* as compared to L*er* (Figure 5A and B). We previously showed that Pol II generates long noncoding transcripts at the *soloLTR* locus [34]. The accumulation of these transcripts at *soloLTR* was also reduced in *top1α-2* (Figure 5B). Therefore, TOP1α contributes to the production of Pol V-dependent or Pol II-dependent long noncoding transcripts.

As the Pol V- or Pol II-dependent long noncoding transcripts facilitate the recruitment of siRNA-AGO4 to chromatin to ultimately result in RdDM or H3K9me2 deposition, we asked whether *TOP1α* promotes AGO4 occupancy at these RdDM target loci. ChIP-qPCR was conducted with anti-Myc antibodies in *Myc-AGO4* [35] and *Myc-AGO4 top1α-2* plants. At four well-known RdDM target loci, AGO4 occupancy was reduced in *top1α-2* (Figure 5C).

To determine whether TOP1α might act directly at these RdDM loci, we examined TOP1α occupancy at these loci. We first generated a *TOP1α-HA* fusion driven by the *TOP1α* promoter (*TOP1α-HA*) and introduced it into *top1α-2*. The morphological phenotypes of *top1α-2* plants were completely rescued by *TOP1α-HA*, indicating that the transgene was functional. We then performed ChIP-qPCR using anti-HA antibodies. TOP1α was found at all six loci examined (Figure 5D).

## Discussion

Beginning with a forward chemical genetics screen with a transcriptionally silenced reporter, *LUCL*, we have discovered that the well-studied anti-cancer compound CPT can de-repress loci undergoing transcriptional silencing by releasing H3K9 methylation and/or DNA methylation. As topoisomerase I is the cellular target of CPT, this implicates topoisomerase I in transcriptional silencing. Indeed, two *top1α* alleles, *top1α-2* and *top1α-7*, mimic CPT treatment in de-repressing the expression of endogenous RdDM target loci and reducing H3K9me2 or DNA methylation levels at these loci.

Here, we first consider whether TOP1α acts through RdDM or independently of RdDM to silence TEs. RdDM requires Pol IV and Pol V, which generate siRNAs and long noncoding RNAs, respectively. We show that TOP1α is dispensable for siRNA accumulation, but is required for the production of Pol V-dependent long noncoding RNAs, which are known to recruit siRNA-AGO4 to chromatin. Consistently, *TOP1α* promotes the recruitment of AGO4 to RdDM target loci. Moreover, 88 out of 97 WT-*top1α* CHH DMRs with reduced methylation in *top1α* also require Pol IV or Pol V for CHH methylation (Figure 3C). *5S* rDNA loci lose CG methylation in *top1α-2* and *nrpe1-11* mutants, and provide an example of a genomic region where CG methylation requires TOP1α, Pol IV, and Pol V. These data suggest that *TOP1α* acts at least in part through RdDM to silence TEs and repeats. However, MethylC-seq analyses revealed that *TOP1α* has a limited role in DNA methylation. We envision two possibilities for the limited role in DNA methylation observed for *TOP1α*. First, *TOP1α* may have a much broader role in DNA methylation in the genome, and the limited effects of *top1α* mutants on DNA methylation could be due to the redundant functions of *TOP1β*. So far, our efforts to knock down *TOP1β* in the *top1α-2* background have been unsuccessful. Second, *TOP1α*'s primary functions may lie in the promotion of H3K9 dimethylation, with DNA methylation being a secondary effect of H3K9 dimethylation. From our studies of a limited number of RdDM loci, we found that reduced H3K9me2 levels, but not necessarily reduced DNA methylation, always accompany the de-repression of these loci by CPT treatment or by mutations in *TOP1α*. Therefore, it is likely that the primary function of *TOP1α* lies in facilitating H3K9me2 deposition. Consistent with this model, the observed effects of CPT treatment on DNA methylation at four loci require *CMT3* and *KYP*, both of which promote H3K9 dimethylation. Another observation consistent with this hypothesis is that CPT treatment had no effect on *LUCH* (Figure 1C), a reporter gene that is strictly repressed by CHH methylation and is insensitive to loss of function in *CMT3* [36]. As *CMT3*-mediated DNA methylation requires H3K9me2 [14], we presume that *LUCH* is not repressed by H3K9me2. The lack of an effect of CPT treatment on *LUCH* would be consistent with TOP1α acting in TGS through H3K9me2 deposition.

Our finding that TOP1α promotes the production of Pol V-dependent transcripts is consistent with what is known about the function of topoisomerases in bacteria and yeast. Topoisomerases are thought to facilitate transcription elongation by relaxing supercoils [37]. Consistent with this model, loss of Top1 in *Schizosaccharomyces pombe* results in the accumulation of Pol II in gene bodies [38], [39]. The parallels of Pol V- and Pol II-mediated transcription have recently been highlighted [40], and we propose that *TOP1α* promotes transcription elongation by Pol V as it does for Pol II.

Although we prefer a model in which TOP1α acts in RdDM by facilitating the production of long noncoding RNAs by Pol V or Pol II, an alternative model cannot be overlooked. Studies in other systems have shown that topoisomerases interact with SMC-containing proteins acting in chromosome compaction [41], [42]. DMS3, a player of the RdDM machinery, contains an SMC domain [43]; therefore, there is a possibility that TOP1α may facilitate RdDM through DMS3.

In summary, we have discovered a role for DNA topoisomerase I in H3K9 methylation and DNA methylation in *Arabidopsis*. Another study showed that chemical inhibitors of topoisomerases I and II release the epigenetic silencing of an imprinted gene in mouse [44]. Together, these studies point to a role of topoisomerases in epigenetic silencing. Given that CPT is a canonical anti-cancer compound and several of its derivatives are presently used in cancer therapy [25], the emerging role of topoisomerase I in epigenetic gene silencing allude to the mode of carcinogenesis.

## Materials and Methods

### MethylC-seq analysis: Identification of Differentially Methylated Regions (DMRs)

Raw data from Illumina sequencing were filtered to remove reads that failed to pass the Illumina quality control and to condense multi-copy reads to a single copy. Hereafter, the reads were mapped to TAIR 10 *Arabidopsis* genome as well as a C-to-T converted genome using BS_Seeker [45] with default settings. Only perfectly and uniquely mapped reads were retained. For L*er* and *top1α*, which are in the Landsberg ecotype, the reads were mapped to a pseudo-L*er* genome generated by incorporating the L*er* polymorphisms into the Tair10 Columbia genome (ftp://ftp.arabidopsis.org/Polymorphisms/Ecker_ler.homozygous_snp.txt). This enables the direct comparison of DMR regions between the Columbia and Landsberg samples.

DMRs were identified following a published method [27] with some modifications. In brief, the genome was split into continuous 100 bp windows. The Cs or Ts were counted in each window in the three different contexts (CG, CHG or CHH) separately. Only windows with least 4 Cs each sequenced at least 4 times in the wild-type sample were kept for the DMR analysis. The methylation level for a window was determined as:
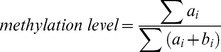
in which *a_i_* denotes the number of read “C”s and *b_i_* denotes the number of read “T”s mapping to the *i*th cytosine site. The methylation level in each window in wild type is then compared to the corresponding window in a mutant. A methylation difference of 0.4, 0.2, and 0.1 for CG, CHG, and CHH, and an adjusted p-value (FDR)<0.01 (Fisher's exact test) were used as the cutoff for defining DMRs.

Additional measures were taken to reduce experimental noise. First, two or three biological replicates/alleles were examined. In deriving initial DMRs, we compared each wild type/mutant pair from the same biological replicate (Table S5). Then, DMRs located within 200 bp of each other were merged. Next, the overlap in DMRs from the two biological replicates/alleles was identified (Table S5). Finally, we removed the DMRs that overlapped with the hypervariability (HV) regions found to be prone to changes in DNA methylation [28], [29] (Table S5).

See Text S1 for Supplemental Methods and Table S8 for oligonucleotides used in this study.

### Accession numbers and data deposition

The gene accession numbers used in this study are At5g55310 (*TOP1α*), At5g55300 (*TOP1β*), At1g05460 (*NRPD1*), and At2g40030 (*NRPE1*). MethylC-seq and small RNA-seq read data have been deposited into NCBI GEO under the identification numbers GSE50691 and GSE50720, respectively.

## Supporting Information

Figure S1 The nature of *top1α* and *top1β* alleles and the effects of *top1α-2* on DNA methylation at several loci. (A) Schematic representation of *TOP1α* and *TOP1β* and several mutant alleles. The white triangles represent T-DNA insertions. *top1α-2* is a point mutation that causes an early stop codon (star). (B) *top1α-2^Col^* does not de-repress *LUCL*. *top1α-2^Col^* is *top1α-2* introgressed into Col-0 through five backcrosses. *LUCL ago4-6* and *LUCL drm2-6* were included as positive controls, as *ago4-6* and *drm2-6* weakly de-repress *LUCL* [20]. (C) DNA blot analysis of the *MEA-ISR* locus. Genomic DNA from ten-day old seedlings was digested with *Msp*I and hybridized with a probe corresponding to the *MEA-ISR* locus. *MspI* cuts unmethylated DNA in a CHG context. The upper and lower bands represent methylated and unmethylated DNA. *nrpe1-11* is a Pol V mutant in the Col-0 background. No change was observed between *top1α-2* and L*er* (the wild-type control for *top1α-2*). (D) DNA blot analysis of 180 bp and 5S repeats. Left panel: Genomic DNA from ten-day old seedlings was digested with *Msp*I and hybridized with a probe corresponding to the 180 bp centromeric repeats. *cmt3-7* is a control with reduced CHG methylation. *top1α-2* has a slight reduction in CHG methylation at the 180 bp repeats as compared to L*er*. Right panel: Genomic DNA from ten-day old seedlings digested with *Hae*III and hybridized with a probe corresponding to the *5S* loci. *Hae*III recognizes the GGCC sequence, but cannot cut when the last C is methylated, thus it is sensitive to CHH methylation. *nrpd1-3* is a Pol IV mutant and *nrpe1-11* is a Pol V mutant. Both serve as controls with reduced CHH methylation and are to be compared to Col-0 as wild type. No change was observed between *top1α-2* and L*er*, the wild-type control for *top1α-2*. (E) CPT treatment results in reductions in DNA methylation at several RdDM loci in a *CMT3*- and *KYP*-dependent manner. McrBC-qPCR analysis was performed to quantify DNA methylation levels in CPT-treated L*er* (wild type), *cmt3-7* or *kyp-2* ten-week-old seedlings. Relative DNA ratios between CPT treatment and DMSO treatment are shown. Higher DNA levels in this assay correlate with lower levels of DNA methylation. *At1g40129* served as an internal unmethylated control. Error bars represent standard deviations calculated from three biological replicates.
(TIF)

Figure S2 Representative screen shots of an overlay of MethylC-seq and small RNA-seq at three genomic regions in various genotypes. The three different loci (A–C) are indicated by their genomic coordinates above the tracks. The top five tracks depict CHH methylation (blue vertical lines). The y-axis indicates the methylation level from 0 (0%) to 1 (100%). The next four tracks represent small RNAs (purple vertical lines). The y-axis indicates small RNA abundance normalized by read depth. The positions of genes or transposable elements (TEs) are indicated below the small RNA tracks, with the green and brown rectangles representing genes and TEs, respectively. The position of these boxes (above or below the line) indicates which DNA strand those features are transcribed from. The black, orange, and purple rectangles at the bottom indicate the positions of WT-*top1α* DMRs, Col-*nrpd1-3* DSRs, and L*er*-*top1α-2* DSRs, respectively. DMRs, differentially methylated regions; DSRs, differential small RNA regions.
(TIF)

Figure S3 *TOP1α* does not globally contribute to small RNA accumulation. (A) Loss of *TOP1α* did not significantly change siRNA (cluster4, soloLTR, siR1003) and miRNA (miR173) levels. RNA blots were performed for L*er* (wild type) and *top1α-2*. U6 was used as an internal loading control. The numbers indicate the relative abundance of the small RNAs in the mutant (with that in the wild type set to 1.0). (B) The size distribution of total small RNA reads in L*er* and *top1α-2* is largely similar. (C) Box-and-whisker plots of global small RNA abundance in various genotypes. The whiskers extend to the most extreme data points that are no more than 1.5 times the interquartile range from the box. Significant reduction is indicated by “*” (P<10^−10^ Mann–Whitney U test). *nrpd1-3* and *nrpe1-11* have mutations in Pol IV and Pol V, respectively, and are to be compared to Col-0 (wild type). Small RNAs were mapped to the genome, which is divided into 500 bp static windows. Only windows in which read abundance was at least 10 RPM in Col-0 or L*er* are considered. The x-axis represents the genotypes as indicated. The y-axis shows normalized read abundance (in RPM, reads per million) in 500 bp windows. Small RNA levels were unaffected in the *top1α* mutant, whereas they were reduced in *nrpd1-3* and *nrpe1-11* as compared to Col-0.
(TIF)

Table S1 Summary of bisulfite conversion efficiency for each genotype.
(PDF)

Table S2 Read coverage of whole genome bisulfite sequencing libraries.
(PDF)

Table S3 Correlation coefficient values for the different biological replicates of each genotype in MethylC-seq.
(PDF)

Table S4 DMRs between wild-type samples.
(PDF)

Table S5 Derivation of DMRs between wild type and *top1α*, *nrpd1-3*, or *nrpe1-11*.
(PDF)

Table S6 Final WT-*top1α* DMRs.
(PDF)

Table S7 Only a small number of differential small RNA regions (DSRs) were found between wild type and *top1α-2*. Whole genome high throughput sequencing was performed for small RNAs in wild type (Col-0 and L*er*), *nrpd1-3*, a Pol IV mutant, *nrpe1-11*, a Pol V mutant, and *top1α-2*. *nrpd1-3* and *nrpe1-11* are to be compared to Col-0 and *top1α-2* is to be compared to L*er*. The genome was divided into 500 bp static windows and small RNA reads in each window were counted and compared between each mutant and its corresponding wild type. Thousands of DSRs were found in *nrpd1-3* or *nrpe1-11* as compared to Col-0, but only 71 were found in *top1α-2* relative to L*er* (see Experimental Procedures for the derivation of DSRs). The numbers of DSRs mapping to different genomic features (TE, gene, and inergenic region) are listed. TE = transposable element. “Reduced” and “increased” refer to DSRs with reduced and increased small RNA read counts in the mutants, respectively.
(PDF)

Table S8 Oligonucleotides used in this study.
(PDF)

Text S1 Supplemental methods.
(DOCX)
