# Correction: DNA Topoisomerase 1α Promotes Transcriptional Silencing of Transposable Elements through DNA Methylation and Histone Lysine 9 Dimethylation in *Arabidopsis*

**DOI:** 10.1371/journal.pgen.1005452

**Published:** 2015-09-17

**Authors:** Thanh Theresa Dinh, Lei Gao, Xigang Liu, Dongming Li, Shengben Li, Yuanyuan Zhao, Michael O'Leary, Brandon Le, Robert J. Schmitz, Pablo A. Manavella, Shaofang Li, Detlef Weigel, Olga Pontes, Joseph R. Ecker, Xuemei Chen

The middle initial of the tenth author’s name is missing. The correct name is: Pablo A. Manavella.
