# Supplementary figures and images for: DNA Topoisomerase 1α Promotes Transcriptional Silencing of Transposable Elements through DNA Methylation and Histone Lysine 9 Dimethylation in Arabidopsis
Source: PLoS Genet. 2014 Jul 3;10(7):e1004446. doi: 10.1371/journal.pgen.1004446 (PMC4080997; doi:10.1371/journal.pgen.1004446)

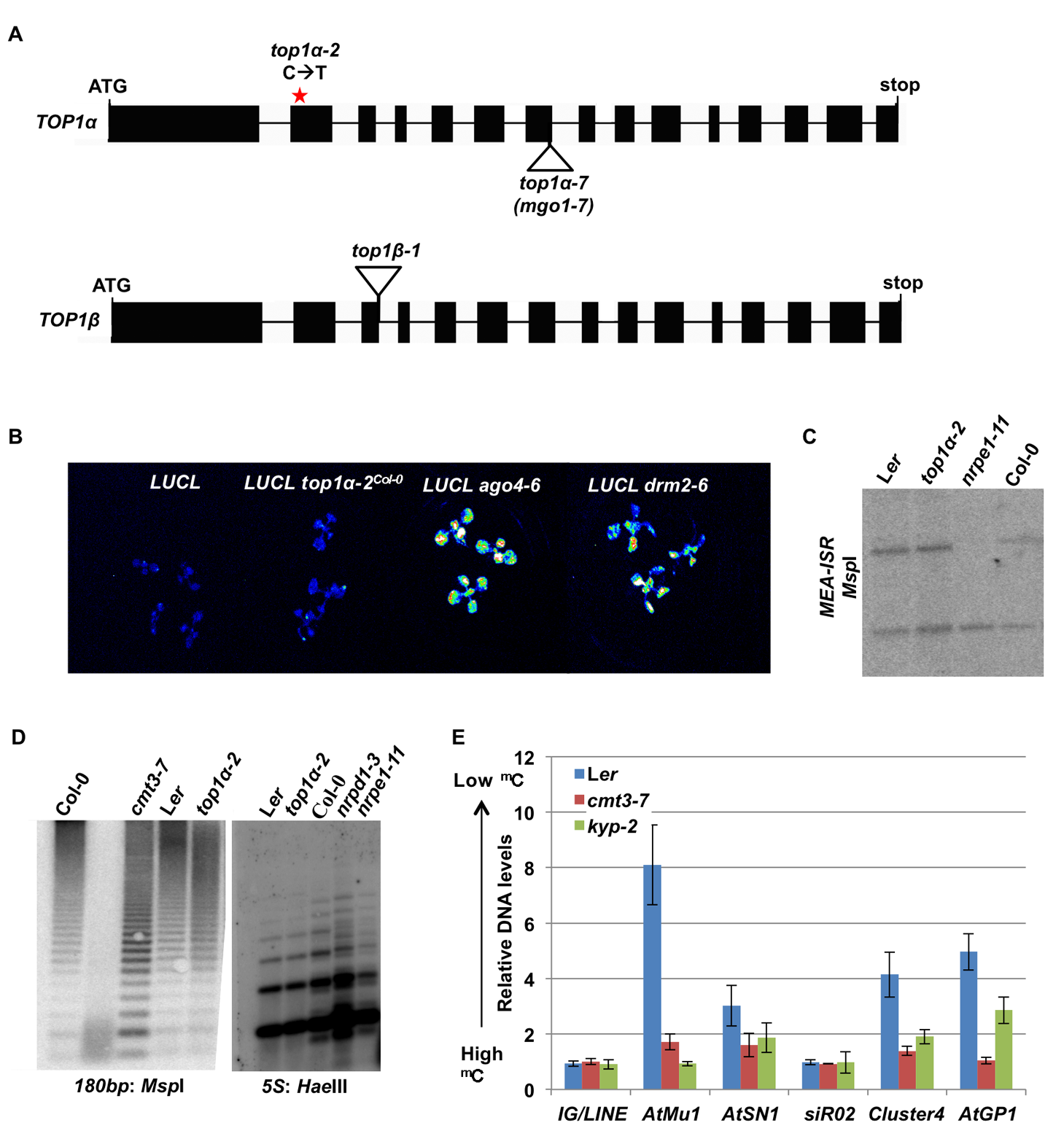

Supplement: Figure S1 — The nature of top1α and top1β alleles and the effects of top1α-2 on DNA methylation at several loci. (A) Schematic representation of TOP1α and TOP1β and several mutant alleles. The white triangles represent T-DNA insertions. top1α-2 is a point mutation that causes an early stop codon (star). (B) top1α-2Col does not de-repress LUCL. top1α-2Col is top1α-2 introgressed into Col-0 through five backcrosses. LUCL ago4-6 and LUCL drm2-6 were included as positive controls, as ago4-6 and drm2-6 weakly de-repress LUCL [20]. (C) DNA blot analysis of the MEA-ISR locus. Genomic DNA from ten-day old seedlings was digested with MspI and hybridized with a probe corresponding to the MEA-ISR locus. MspI cuts unmethylated DNA in a CHG context. The upper and lower bands represent methylated and unmethylated DNA. nrpe1-11 is a Pol V mutant in the Col-0 background. No change was observed between top1α-2 and Ler (the wild-type control for top1α-2). (D) DNA blot analysis of 180 bp and 5S repeats. Left panel: Genomic DNA from ten-day old seedlings was digested with MspI and hybridized with a probe corresponding to the 180 bp centromeric repeats. cmt3-7 is a control with reduced CHG methylation. top1α-2 has a slight reduction in CHG methylation at the 180 bp repeats as compared to Ler. Right panel: Genomic DNA from ten-day old seedlings digested with HaeIII and hybridized with a probe corresponding to the 5S loci. HaeIII recognizes the GGCC sequence, but cannot cut when the last C is methylated, thus it is sensitive to CHH methylation. nrpd1-3 is a Pol IV mutant and nrpe1-11 is a Pol V mutant. Both serve as controls with reduced CHH methylation and are to be compared to Col-0 as wild type. No change was observed between top1α-2 and Ler, the wild-type control for top1α-2. (E) CPT treatment results in reductions in DNA methylation at several RdDM loci in a CMT3- and KYP-dependent manner. McrBC-qPCR analysis was performed to quantify DNA methylation levels in CPT-treated Ler (wild type), cmt3-7 [file pgen.1004446.s001.tif]

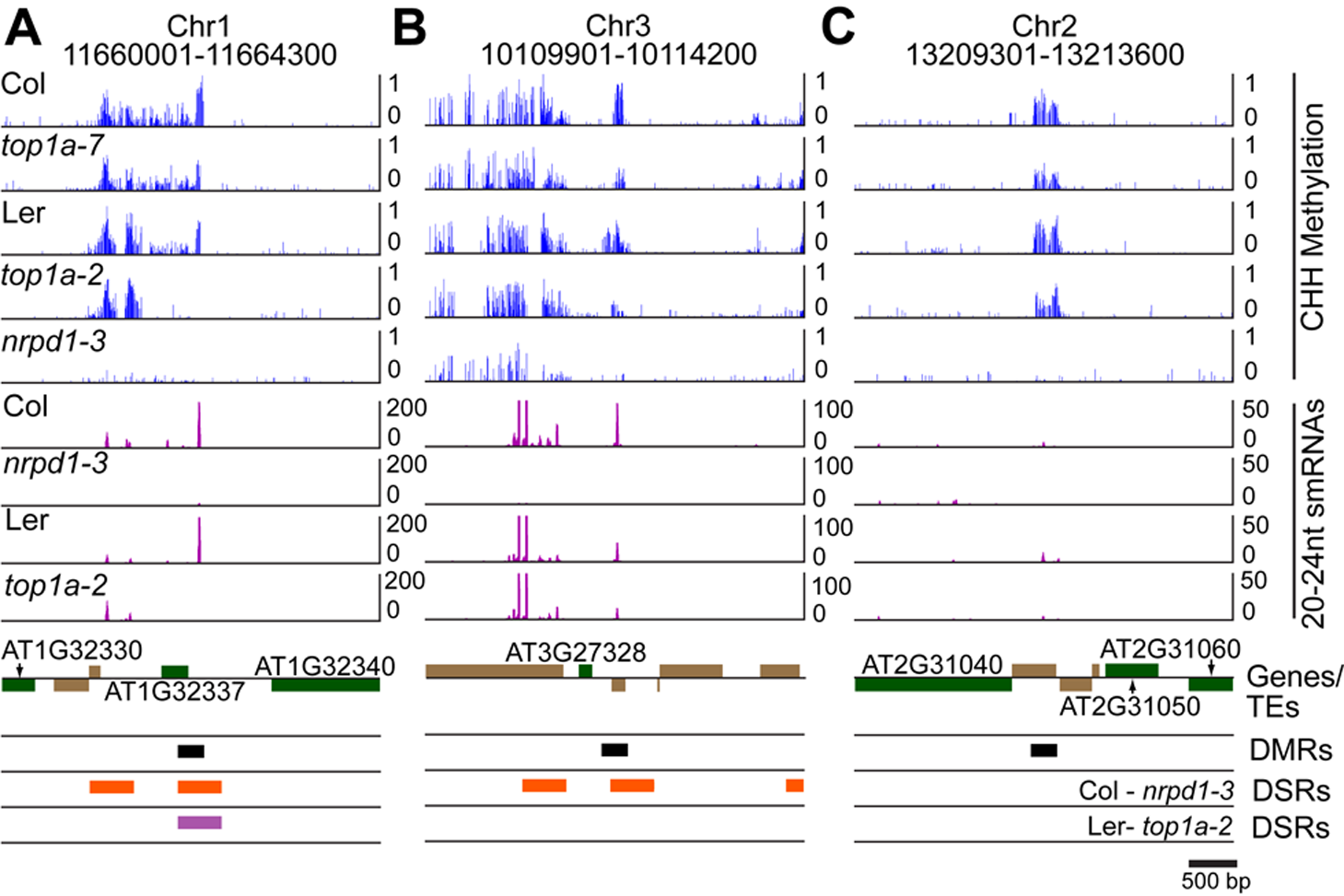

Supplement: Figure S2 — Representative screen shots of an overlay of MethylC-seq and small RNA-seq at three genomic regions in various genotypes. The three different loci (A–C) are indicated by their genomic coordinates above the tracks. The top five tracks depict CHH methylation (blue vertical lines). The y-axis indicates the methylation level from 0 (0%) to 1 (100%). The next four tracks represent small RNAs (purple vertical lines). The y-axis indicates small RNA abundance normalized by read depth. The positions of genes or transposable elements (TEs) are indicated below the small RNA tracks, with the green and brown rectangles representing genes and TEs, respectively. The position of these boxes (above or below the line) indicates which DNA strand those features are transcribed from. The black, orange, and purple rectangles at the bottom indicate the positions of WT-top1α DMRs, Col-nrpd1-3 DSRs, and Ler-top1α-2 DSRs, respectively. DMRs, differentially methylated regions; DSRs, differential small RNA regions. (TIF) [file pgen.1004446.s002.tif]

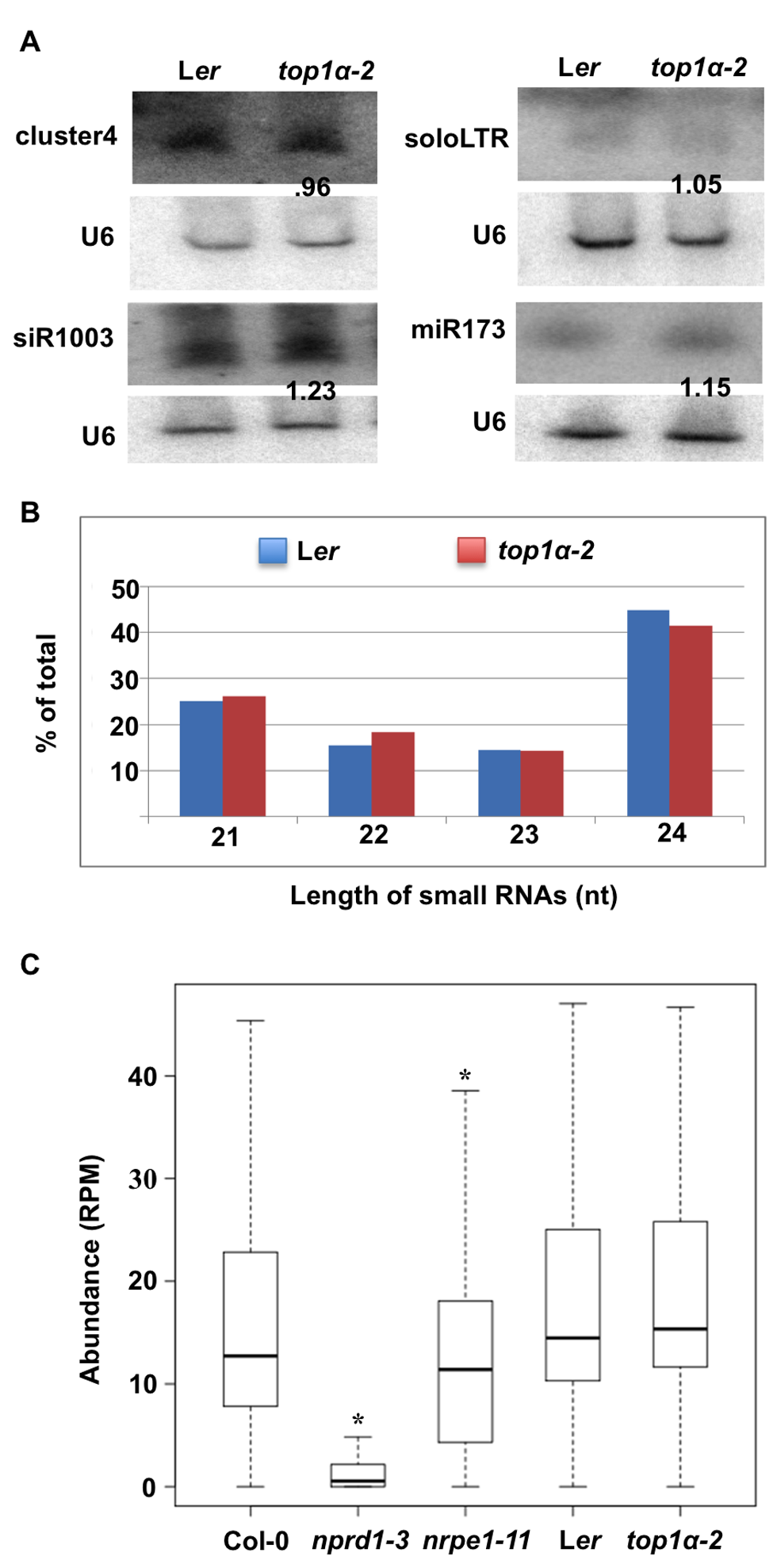

Supplement: Figure S3 — TOP1α does not globally contribute to small RNA accumulation. (A) Loss of TOP1α did not significantly change siRNA (cluster4, soloLTR, siR1003) and miRNA (miR173) levels. RNA blots were performed for Ler (wild type) and top1α-2. U6 was used as an internal loading control. The numbers indicate the relative abundance of the small RNAs in the mutant (with that in the wild type set to 1.0). (B) The size distribution of total small RNA reads in Ler and top1α-2 is largely similar. (C) Box-and-whisker plots of global small RNA abundance in various genotypes. The whiskers extend to the most extreme data points that are no more than 1.5 times the interquartile range from the box. Significant reduction is indicated by “*” (P<10−10 Mann–Whitney U test). nrpd1-3 and nrpe1-11 have mutations in Pol IV and Pol V, respectively, and are to be compared to Col-0 (wild type). Small RNAs were mapped to the genome, which is divided into 500 bp static windows. Only windows in which read abundance was at least 10 RPM in Col-0 or Ler are considered. The x-axis represents the genotypes as indicated. The y-axis shows normalized read abundance (in RPM, reads per million) in 500 bp windows. Small RNA levels were unaffected in the top1α mutant, whereas they were reduced in nrpd1-3 and nrpe1-11 as compared to Col-0. (TIF) [file pgen.1004446.s003.tif]
