# Supplementary material for: DNA Topoisomerase 1α Promotes Transcriptional Silencing of Transposable Elements through DNA Methylation and Histone Lysine 9 Dimethylation in Arabidopsis
Source: PLoS Genet. 2014 Jul 3;10(7):e1004446. doi: 10.1371/journal.pgen.1004446 (PMC4080997; doi:10.1371/journal.pgen.1004446)
Supplement: Table S1 — Summary of bisulfite conversion efficiency for each genotype. (PDF) [file pgen.1004446.s004.pdf]

Table S1. Summary of bisulfite conversion efficiency for each genotype

	**CG**	**CHG**	**CHH**	**Total C**
**Col-0 A***	98.0%	97.9%	97.8%	97.8%
**Col-0 B***	98.2%	98.1%	97.8%	97.9%
**Col-0 C***	98.0%	97.9%	97.8%	97.9%
***nrpd1-3* B**	98.0%	98.0%	97.8%	97.9%
***nrpd1-3* C**	97.9%	97.8%	97.7%	97.7%
***nrpe1-11* B**	98.1%	98.1%	97.9%	97.9%
***nrpe1-11* C**	98.0%	98.0%	97.9%	97.9%
***top1α-7* A**	98.0%	97.9%	97.8%	97.9%
**L*er* A**	97.7%	97.7%	97.6%	97.6%
***top1α-2* A**	97.6%	97.6%	97.5%	97.5%

* "A", "B", and "C" denote different biological replicates. All samples with the same letter notation were processed at the same time with the biological materials grown at the same time and in the same manner.
