# Supplementary material for: DNA Topoisomerase 1α Promotes Transcriptional Silencing of Transposable Elements through DNA Methylation and Histone Lysine 9 Dimethylation in Arabidopsis
Source: PLoS Genet. 2014 Jul 3;10(7):e1004446. doi: 10.1371/journal.pgen.1004446 (PMC4080997; doi:10.1371/journal.pgen.1004446)
Supplement: Table S2 — Read coverage of whole genome bisulfite sequencing libraries. (PDF) [file pgen.1004446.s005.pdf]

Table S2. Read coverage of whole genome bisulfite sequencing libraries

CHH	# of sequenced ^{m}C	# of total sequenced C	31198380 *
			Coverage[&]
Col-0 A[§]	12949522	351132278	11.255
Col-0 B[§]	6974394	228191510	7.314
Col-0 C[§]	8081587	276670598	8.868
top1α-7 A	10469168	316538238	10.146
L*er* A	11536699	291162622	9.333
top1α-2 A	11304607	298325796	9.562
nrpd1-3 B	3996254	221784087	7.109
nrpd1-3 C	3928539	238723704	7.652
nrpe1-11 B	5488089	293648870	9.412
nrpe1-11 C	3147078	175298018	5.619
CG	# of sequenced ^{m}C	# of total sequenced C	5567714 **
			Coverage[&]
Col-0 A	20359074	63064636	11.327
Col-0 B	11967876	40092653	7.201
Col-0 C	14292137	48943906	8.791
top1α-7 A	17407808	57735189	10.370
L*er* A	16292750	54895136	9.860
top1α-2 A	15579371	55729079	10.009
nrpd1-3 B	11233072	40655236	7.302
nrpd1-3 C	11410979	42322601	7.601
nrpe1-11 B	14992909	51859716	9.314
nrpe1-11 C	8591198	30902224	5.550
CHG	# of sequenced ^{m}C	# of total sequenced C	6093657 ***
			Coverage[&]
Col-0 B	7756135	66779144	10.959
Col-0 A	4368090	43083879	7.070
Col-0 C	5122123	52159761	8.560
top1α-7 A	6866937	61918007	10.161
L*er* A	6504312	59145337	9.706
top1α-2 A	6227381	60355438	9.905
nrpd1-3 B	3536815	43885788	7.202
nrpd1-3 C	3458560	45355541	7.443
nrpe1-11 B	4982617	55895446	9.173
nrpe1-11 C	2750976	33011274	5.417
Total	# of sequenced ^{m}C	# of total sequenced C	42859751 ****
			Coverage[&]
Col-0 B	41064731	480976058	11.222
Col-0 A	23310360	311368042	7.265

**Col-0 C**	27495847	377774265	8.814
***top1α-7* A**	34743913	436191434	10.177
**L*er* A**	34333761	405203095	9.454
***top1α-2* A**	33111359	414410313	9.669
***nrpd1-3* B**	18766141	306325111	7.147
***nrpd1-3* C**	18798078	326401846	7.616
***nrpe1-11* B**	25463615	401404032	9.366
***nrpe1-11* C**	14489252	239211516	5.581

* # of total CHH sites in genome ** # of total CG sites in genome *** # of total CHG sites in genome **** # of total C sites in genome

[&] Coverage = # of total sequenced C/# of total CXX sites in genome

[§] "A", "B", and "C" denote different biological replicates. All samples with the same letter notation were processed at the same time with the biological materials grown at the same time and in the same manner.
