# Supplementary material for: DNA Topoisomerase 1α Promotes Transcriptional Silencing of Transposable Elements through DNA Methylation and Histone Lysine 9 Dimethylation in Arabidopsis
Source: PLoS Genet. 2014 Jul 3;10(7):e1004446. doi: 10.1371/journal.pgen.1004446 (PMC4080997; doi:10.1371/journal.pgen.1004446)
Supplement: Table S3 — Correlation coefficient values for the different biological replicates of each genotype in MethylC-seq. (PDF) [file pgen.1004446.s006.pdf]

Table S3. Correlation coefficient values for the different biological replicates of each genotype in MethylC-seq

	**Col-0 A**	**Col-0 B**	**Col-0 C**
**Col-0 A**[§]		0.997	0.997
**Col-0 B**[§]	0.997		0.997
**Col-0 C**[§]	0.997	0.997	
	***nrpd1-3* C**		
***nrpd1-3* B**	0.998		
	***nrpe1-11* C**		
nrpe1-11 B	0.997		

[§] "A", "B", and "C" denote different biological replicates. All samples with the same letter notation were processed at the same time with the biological materials grown at the same time and in the same manner.
