# Supplementary material for: DNA Topoisomerase 1α Promotes Transcriptional Silencing of Transposable Elements through DNA Methylation and Histone Lysine 9 Dimethylation in Arabidopsis
Source: PLoS Genet. 2014 Jul 3;10(7):e1004446. doi: 10.1371/journal.pgen.1004446 (PMC4080997; doi:10.1371/journal.pgen.1004446)
Supplement: Table S4 — DMRs between wild-type samples. (PDF) [file pgen.1004446.s007.pdf]

Table S4. DMRs between wild-type samples

A. Number of DMRs between any two Col replicates in this study

	CG	CHG	CHH
Col A vs. Col B	21	8	2139
Col A vs. Col C	66	19	3452
Col B vs. Col C	10	2	1708

B. Number of DMRs between any two wild-type (WT) samples in a published study [27]

	CG	CHG	CHH
WT1 vs. WT2	1978	2804	15393
WT1 vs. WT3	1762	213	4151
WT2 vs. WT3	446	550	15392

27. Stroud H, Greenberg MV, Feng S, Bernatavichute YV, Jacobsen SE (2013) Comprehensive analysis of silencing mutants reveals complex regulation of the *Arabidopsis* methylome. Cell 152: 352-364.
