# Supplementary material for: DNA Topoisomerase 1α Promotes Transcriptional Silencing of Transposable Elements through DNA Methylation and Histone Lysine 9 Dimethylation in Arabidopsis
Source: PLoS Genet. 2014 Jul 3;10(7):e1004446. doi: 10.1371/journal.pgen.1004446 (PMC4080997; doi:10.1371/journal.pgen.1004446)
Supplement: Table S5 — Derivation of DMRs between wild type and top1α, nrpd1-3, or nrpe1-11. (PDF) [file pgen.1004446.s008.pdf]

Table S5. Derivation of DMRs between wild type and *top1α*, *nrpd1-3*, or *nrpe1-11*

A. DMRs between wild type and *top1α*

Col A vs. *top1α-7* A*

100 bp DMRs in *top1α-7*

	CHH	CG	CHG
increased^	1151	1525	147
reduced^	3143	2378	85

Merge the DMRs that lie within 200bp of each other

	CHH	CG	CHG
increased	1080	1421	113
reduced	2722	2190	65

Ler A vs. *top1α-2* A*

100 bp DMRs in *top1α-2*

	CHH	CG	CHG
increased	768	738	106
reduced	1357	1848	505

Merge the DMRs that lie within 200bp of each other

	CHH	CG	CHG
increased	719	699	85
reduced	1029	1545	270

Overlap between *top1α-7* and *top1α-2*

	CHH	CG	CHG
increased	11	12	1
reduced	98	36	0

Final DMRs after removing overlap with HVR**

	CHH	CG	CHG
increased	10	9	1
reduced	97	35	0

B. DMRs between wild type (Col) and *nrpd1-3*

Col B vs. *nrpd1-3* B*

100 bp DMRs in *nrpd1-3* B

	CHH	CG	CHG
increased^	307	2708	198
reduced^	27892	3982	7928

Merge the DMRs that lie within 200bp of each other

	CHH	CG	CHG
increased	290	2532	146
reduced	10465	3330	5525

Col C vs. *nrpd1-3* C*

100 bp DMRs in *nrpd1-3* C

	CHH	CG	CHG
increased	329	3465	311
reduced	28583	4415	8914

Merge the DMRs that lie within 200bp of each other

	CHH	CG	CHG
	313	3192	227
	10629	3704	6067

Overlap between *nrpd1-3* B and *nrpd1-3* C

	CHH	CG	CHG
increased	10	1413	57
reduced	8234	1981	4442

Final DMRs after removing overlap with HVR**

	CHH	CG	CHG
increased	9	1251	42
reduced	7541	1850	4319

C. DMRs between wild type (Col) and *nrpe1-11*

Col B vs. *nrpe1-11* B*

100 bp DMRs in *nrpe1-11* B

	CHH	CG	CHG

Col C vs. *nrpe1-11* C*

100 bp DMRs in *nrpe1-11* C

	CHH	CG	CHG

**increased^**	446	3670	548
**reduced^**	30049	5842	9418

**increased**	639	4205	442
**reduced**	26762	5893	8163

Merge the DMRs that lie within 200bp of each other

	CHH	CG	CHG
**increased**	418	3423	424
**reduced**	10587	4696	6261

Merge the DMRs that lie within 200bp of each other

	CHH	CG	CHG
**increased**	608	3859	339
**reduced**	9966	4655	5598

Overlap between *nrpe1-11* B and *nrpe1-11* C

	CHH	CG	CHG
**increased**	16	1952	131
**reduced**	8209	3258	4815

Final DMRs after removing overlap with HVR**

	CHH	CG	CHG
**increased**	15	1714	107
**reduced**	7535	3091	4677

* “A”, “B”, and “C” denote different biological replicates. All samples with the same letter notation were processed at the same time with the biological materials grown at the same time and in the same manner.

**** Hypervariable Regions (HVR)**
HVR regions were according to studies by Becker et al. (2011) Nature 480, 245-249 and Schmitz et al. (2011) Science 334, 369-373.
Final DMR= Overlap of two alleles or replicates - Overlap with HVR

^ "increased" and "reduced" refer to DMRs with increased and reduced DNA methylation in the mutant relative to wild type, respectively.
