# Supplementary material for: DNA Topoisomerase 1α Promotes Transcriptional Silencing of Transposable Elements through DNA Methylation and Histone Lysine 9 Dimethylation in Arabidopsis
Source: PLoS Genet. 2014 Jul 3;10(7):e1004446. doi: 10.1371/journal.pgen.1004446 (PMC4080997; doi:10.1371/journal.pgen.1004446)
Supplement: Table S6 — Final WT-top1α DMRs. (PDF) [file pgen.1004446.s009.pdf]

Table S6. Final WT-*top1α* DMRs

reduced		
**CG**	**CHG**	**CHH**
Chr2_11502500_600		Chr1_11662000_300
Chr2_12614200_100		Chr1_15940400_100
Chr2_16728400_100		Chr1_16645900_100
Chr3_1473600_100		Chr1_17293300_100
Chr3_17608000_100		Chr1_19986900_100
Chr5_20159600_100		Chr1_20565000_100
Chr5_23506200_100		Chr1_21582200_200
Chr5_6398500_400		Chr1_23068200_300
Chr5_893200_100		Chr1_23312900_300
Chr1_18403800_500		Chr1_3980700_400
Chr3_8560700_100		Chr1_5056000_200
Chr5_22497600_400		Chr1_56400_200
Chr3_18134900_100		Chr1_6273500_200
Chr3_8977400_1000		Chr1_7936600_200
Chr4_4876400_100		Chr1_9811700_400
Chr1_1007200_100		Chr1_9852800_100
Chr1_13106800_100		Chr2_11160900_200
Chr1_17295200_100		Chr2_13211300_300
Chr1_17322300_200		Chr2_14846600_100
Chr1_17448300_100		Chr2_15402100_100
Chr1_20717000_100		Chr2_16007800_300
Chr1_24910200_100		Chr2_16021700_100
Chr1_25598000_400		Chr2_16273100_100
Chr1_3429300_100		Chr2_16834400_100
Chr1_4453000_100		Chr2_1709600_200
Chr3_1675800_100		Chr2_6390500_200
Chr3_18756100_300		Chr2_7178500_100
Chr3_53500_100		Chr2_7889700_100
Chr3_5690900_100		Chr2_8017600_100
Chr4_15370300_400		Chr2_8575400_400
Chr4_568600_200		Chr2_904600_600
Chr4_6542600_100		Chr3_10111900_300
Chr5_16160300_100		Chr3_10293300_100
Chr5_16393100_100		Chr3_10461000_500
Chr5_21517800_100		Chr3_10827300_100
		Chr3_11099200_500
		Chr3_11279400_200
		Chr3_11374900_100
		Chr3_15654700_100
		Chr3_15741300_100
		Chr3_15915900_100
		Chr3_16395700_200
		Chr3_18412300_100
		Chr3_18652100_200
		Chr3_18758100_200
		Chr3_18896000_200
		Chr3_19291200_100
		Chr3_20330200_100
		Chr3_21608500_200
		Chr3_3838600_200
		Chr3_4745700_100
		Chr3_7981200_100
		Chr3_7981800_100

increased		
**CG**	**CHG**	**CHH**
Chr1_15925200_100	Chr2_6147000_100	Chr1_1293500_100
Chr4_7724400_200		Chr1_13313800_200
Chr3_5365500_100		Chr1_9384800_100
Chr3_8352000_100		Chr2_19414400_100
Chr1_7093000_100		Chr2_6677600_100
Chr2_13238100_100		Chr2_7961300_100
Chr2_13358600_100		Chr3_11501800_200
Chr3_11722000_100		Chr3_15729200_100
Chr3_8523700_100		Chr5_10232300_100
		Chr5_9340100_100

Key:
Pol IV- &Pol V-dependent
Pol IV-dependent
Pol V-dependent
Pol IV- &Pol V-independent

The DMRs are denoted by their chromosome locations and the positions of their first nucleotides and the lengths of the regions.

Chr3_8397400_200
Chr3_9853000_100
Chr4_1064100_100
Chr4_17213900_100
Chr4_17624600_500
Chr4_2367300_100
Chr4_2398900_300
Chr4_298000_100
Chr4_3355100_100
Chr4_5144000_100
Chr4_5559700_100
Chr4_8013200_100
Chr4_8252300_200
Chr5_10531200_100
Chr5_11030500_300
Chr5_13723200_100
Chr5_15012300_100
Chr5_15942300_300
Chr5_18624800_100
Chr5_19526300_300
Chr5_20209300_400
Chr5_20427400_200
Chr5_20527500_400
Chr5_20743700_100
Chr5_20855700_100
Chr5_21028100_100
Chr5_22621300_100
Chr5_24370800_200
Chr5_7467800_300
Chr5_8037400_100
Chr5_8101400_100
Chr5_8160600_400
Chr5_8893300_100
Chr4_2416000_300
Chr3_14241100_100
Chr1_12016000_100
Chr1_15935500_200
Chr2_17190200_100
Chr2_6853000_100
Chr2_8092100_100
Chr3_16647200_200
Chr3_8328700_100
Chr5_12399200_100
Chr5_9642100_100
