# Supplementary material for: DNA Topoisomerase 1α Promotes Transcriptional Silencing of Transposable Elements through DNA Methylation and Histone Lysine 9 Dimethylation in Arabidopsis
Source: PLoS Genet. 2014 Jul 3;10(7):e1004446. doi: 10.1371/journal.pgen.1004446 (PMC4080997; doi:10.1371/journal.pgen.1004446)
Supplement: Table S7 — Only a small number of differential small RNA regions (DSRs) were found between wild type and top1α-2. Whole genome high throughput sequencing was performed for small RNAs in wild type (Col-0 and Ler), nrpd1-3, a Pol IV mutant, nrpe1-11, a Pol V mutant, and top1α-2. nrpd1-3 and nrpe1-11 are to be compared to Col-0 and top1α-2 is to be compared to Ler. The genome was divided into 500 bp static windows and small RNA reads in each window were counted and compared between each mutant and its corresponding wild type. Thousands of DSRs were found in nrpd1-3 or nrpe1-11 as compared to Col-0, but only 71 were found in top1α-2 relative to Ler (see Experimental Procedures for the derivation of DSRs). The numbers of DSRs mapping to different genomic features (TE, gene, and inergenic region) are listed. TE = transposable element. “Reduced” and “increased” refer to DSRs with reduced and increased small RNA read counts in the mutants, respectively. (PDF) [file pgen.1004446.s010.pdf]

Table S7. Only a small number of differential small RNA regions (DSRs) were found between wild type and *top1α-2*.

Whole genome high throughput sequencing was performed for small RNAs in wild type (Col-0 and L*er*), *nrpd1-3,* a Pol IV mutant, *nrpe1-11*, a Pol V mutant, and *top1α-2*. *nrpd1-3* and *nrpe1-11* are to be compared to Col-0 and *top1α-2* is to be compared to L*er*. The genome was divided into 500bp static windows and small RNA reads in each window were counted and compared between each mutant and its corresponding wild type. Thousands of DSRs were found in *nrpd1-3* or *nrpe1-11* as compared to Col-0, but only 71 were found in *top1α-2* relative to L*er* (see Experimental Procedures for the derivation of DSRs). The numbers of DSRs mapping to different genomic features (TE, gene, and inergenic region) are listed. TE= transposable element. "Reduced" and "increased" refer to DSRs with reduced and increased small RNA read count in the mutants, respectively.

	***nrpd1-3*/Col-0**	***nrpe1-11*/Col-0**	***top1α-2*/L*er***
**>=10 RPM***	10729	13961	10066
**reduced**	***nrpd1-3*/Col-0**	***nrpe1-11*/Col-0**	***top1α-2*/L*er***
**TE**	5746	2089	25
**gene**	681	352	15
**intergenic**	2056	1083	31
**total**	8483	3524	71
**increased**	***nrpd1-3*/Col-0**	***nrpe1-11*/Col-0**	***top1α-2*/L*er***
**TE**	9	294	106
**gene**	27	31	20
**intergenic**	14	60	17
**total**	50	385	143

* The numbers of windows with at least 10 RPM (reads per million) of small RNAs in one of the two libraries. Only these windows were used in subsequent DSR derivation.
