# Supplementary material for: DNA Topoisomerase 1α Promotes Transcriptional Silencing of Transposable Elements through DNA Methylation and Histone Lysine 9 Dimethylation in Arabidopsis
Source: PLoS Genet. 2014 Jul 3;10(7):e1004446. doi: 10.1371/journal.pgen.1004446 (PMC4080997; doi:10.1371/journal.pgen.1004446)
Supplement: Table S8 — Oligonucleotides used in this study. (PDF) [file pgen.1004446.s011.pdf]

Table S8. Oligonucleotides used in this study

Name	Oligonucleotide Sequence	Application
**lucp6**	5'-GCACCCGGGGAAGACGCCAAAAACATAAAGAAA-3'	McrBC-PCR, Southern blot
**lucp7**	5'-GGACCCGGGTGCGATCTTTCCGCCCTTCTTGGCCT-3'	McrBC-PCR, Southern blot
**Actin1-F**	5'-CCAAGCAGCATGAAGATCAA-3'	McrBC-PCR
**Actin1-R**	5'-TGAACAATCGATGGACCTGA-3'	McrBC-PCR
**35Sf**	5'-CAAAGCAAGTGGATTGATGTGA-3'	McrBC-PCR, Southern blot
**35Sr**	5'-TTTCCACGATGCTCCTCGT-3'	Southern blot
**LUC 0.13k R**	5'-TATGTGCATCTGTAAAAGCAA-3'	McrBC-PCR
**YZ 35S Bis F**	5'-AttAtTGTyGGtAGAGGtATtTTGAAyGATAGtt-3'	Bisulfite sequencing
**YZ LUC Bis R**	5'-CATCTaTAAAAaCAATTaTTCCAaaAACCAaa-3'	Bisulfite sequencing
**N_UBQ5**	5'-GGTGCTAAGAAGAGGAAGAAT-3'	RT-PCR, loading control
**C_UBQ5**	5'-CTCCTTCTTTCTGGTAAACGT-3'	RT-PCR, loading control
**LUCmF5**	5'-CTCCCCTCTCTAAGGAAGTCG-3'	RT-PCR for LUC
**LUCmR5**	5'-CCAGAATGTAGCCATCCATC-3'	RT-PCR for LUC
**At2g19990-F**	5'-TCACCCGAACAGTTGGAAGAA-3'	McrBC-PCR
**At2g19990-R**	5'-GTGAGGAACCGGTCCATTATTGCT-3'	McrBC-PCR
**Cluster4-F1**	5'-CGTCCTCAAAGTTCCAGAGAT -3'	qRT-PCR

**Cluster4-R1**	5'-CGGTATTCTCCATCCCAAAG -3'	qRT-PCR
**AtCopia2-F1**	5'-TTGCCCCAACAACAAAAA -3'	qRT-PCR
**AtCopia2-R1**	5'-CAGAGAAAGAGATAGAAGAAATGA -3'	qRT-PCR
**AtMuI-F1**	5'-GGCAGTCGGTTTGTCATTCT -3'	qRT-PCR
**AtMuI-R1**	5'-CCTTCTTGGCATGGTTCTTC -3'	qRT-PCR
**MEA ISR-F1**	5'-CGCGAACGACTATTGCTAAA -3'	qRT-PCR B region
**MEA ISR-R1**	5'-ACGATTCCACAAATCCAACA-3'	qRT-PCR B region
**MEA-RT-R**	5'-TGAAATCTAACCGGATTTTGG -3'	Gene specific primer for RT B region
**LNA-siR1003**	5'-A+TGC+CAA+GTT+TGG+CCT+CAC+CGT+C-3'	Probe for northern blot
**LNA-cluster4**	5'-AA+GATC+AAAC+ATCA+GCA+GCGTC+AG+AGG+CTT-3'	Probe for northern blot
**SoloLTR**	5'-GGATTCACGATTAGAGAACGTAGA-3'	Probe for northern blot
**LNA-miR173**	5'-GT+GAT+TTC+TCT+CTG+TAA+GCG+AA-3'	Probe for northern blot
**ACTIN60-F**	5'-ATCCCTCAGCACCTTCCAAC-3'	qRT-PCR; control
**ACTIN60-R**	5'-AAAATCCACATAACAACAGATAGTTCA-3'	qRT-PCR; control
**Chloro_up_bis F**	5'-TATGGTGAGYTACAATAATGGTTAAAGAG-3'	Bisulfite sequencing
**Chloro_up_bis R**	5'-TATCTTTACCRATTAACCAATTTCTAAAC-3'	Bisulfite sequencing
**IGN24-F**	5'-CGCATACGATGGTCGGAGAGTT -3'	RT-PCR B region
**IGN24-R**	5'-GCTTATCATTATCCAAACTTGATCCTATCCTAAA -3'	RT-PCR B region
**AtSN1-F**	5’-CCAGAAATTCATCTTCTTTGGAAAAG-3’	RT-PCR B region
**AtSN1-R**	5’-GCCCAGTGGTAAATCTCTCAGATAGA-3’	RT-PCR B

		region
**IGN26-F**	5'-CTCTTTCAGTGCGACAGCCTCAT-3'	RT-PCR B region
**IGN26-R**	5'-CGGCCAGGAAACCCTAACTTCC-3'	RT-PCR B region
**IGN5-F**	5'-CGCAGCGGAATTGACATCCTATC-3'	RT-PCR B region
**IGN5-R**	5'-TCGGAAAGAGACTCTCCGCTAGAAA-3'	RT-PCR B region
**IGN25-F**	5'-CTTCTTATCGTGTTACATTGAGAACTCTTTCC-3'	RT-PCR B region
**IGN25-R**	5'-ATTCGTGTGGGCTTGGCCTCTT-3'	RT-PCR B region
**IGN15-F**	5'-CCATAGCATAGAAACTTGGCGATATATGAA-3'	RT-PCR B region
**IGN15-R**	5'-CGGAAAAGGTAAGGTGGTTGGAAAA-3'	RT-PCR B region
**IGN23-F**	5'-ACTGAAAATTGTAAACAAAGAAACGGCACTACA-3'	RT-PCR B region
**IGN23-R**	5'-GATCGGTCCATAAACTTGTTGGGTTT-3'	RT-PCR B region
**AtSN1-F**	5'-ACCAACGTGCTGTTGGCCCAGTGGTAAATC-3'	qRT-PCR A region; ChIP-qPCR; McrBC-qPCR
**AtSN1-R**	5'-AAAATAAGTGGTGGTTGTACAAGC-3'	qRT-PCR A region; ChIP-qPCR; McrBC-qPCR
**AtMu1-F1**	5'-CCGAGAACTGGTTGTGGTTT-3'	qRT-PCR A region; ChIP-qPCR; McrBC-qPCR
**AtMu1-R1**	5'-GCTCTTGCTTTGGTGATGGT-3'	qRT-PCR A region; ChIP-qPCR; McrBC-qPCR
**IGN5-F**	5'-AAGCCCAAACCATACACTAATAATCTAAT-3'	ChIP-qPCR
**IGN5-R**	5'-CCGAATAACAGCAAGTCCTTTTAATA-3'	ChIP-qPCR

**IR-71 F1**	5'-TATCATCCTTCTGGTTTTGG-3'	qRT-PCR A region; McrBC-qPCR
**IR-71 R1**	5'-AAGCAACATTCATTTCAGC-3'	qRT-PCR A region; McrBC-qPCR
**siR02-F2**	5'-CAATATGTTCTTCACCATCG-3'	qRT-PCR A region; McrBC-qPCR
**siR02-R2**	5'-ATTTGCGAAACTAATGGAAG-3'	qRT-PCR A region; McrBC-qPCR
**cluster4-F**	5'-CGTCCTCAAAGTTCCAGA-3'	qRT-PCR A region; McrBC-qPCR
**cluster4-R**	5'-GGTATTCTCCATCCCAAAG-3'	qRT-PCR A region; McrBC-qPCR
**SoloLTR;IG/LINE-F**	5'-AACTAACGTCATTACATACACATCTTG-3'	ChIP-qPCR
**SoloLTR;IG/LINE-R**	5'-AATTAGGATCTTGTTTGCCAGCTA-3'	ChIP-qPCR
**AtGP1-F**	5'-TGGTTTTTCCTGTCCAGTTTG-3'	qRT-PCR A region; ChIP-qPCR; McrBC-qPCR
**AtGP1-R**	5'-AACAATCCTAACCGGGTTCC-3'	qRT-PCR A region; ChIP-qPCR; McrBC-qPCR
**EIF4A1-F**	5'-TCTTGGTGAAGCGTGATGAG-3'	ChIP-qPCR
**EIF4A1-R**	5'-GCTGAGTTGGGAGATCGAAG-3'	ChIP-qPCR
**chr2_1882324_CHH-F**	5'-CGAGCCAAAAATTGTTGAAAT-3'	McrBC-qPCR

**chr2_1882324_CHH-R**	5'-TTTGAGGGGATAGAGTATCTCTTG-3'	McrBC-qPCR
**TOP1αgeno-F**	5'-CACCGGCAAGACGGATCTGGAGGG-3'	Generation of *TOP1α-HA*
**TOP1αgeno-R**	5'-GGGTAAACGAGATCGTTGAATGAC-3'	Generation of *TOP1α-HA*
