# Supplementary material for: DNA Topoisomerase 1α Promotes Transcriptional Silencing of Transposable Elements through DNA Methylation and Histone Lysine 9 Dimethylation in Arabidopsis
Source: PLoS Genet. 2014 Jul 3;10(7):e1004446. doi: 10.1371/journal.pgen.1004446 (PMC4080997; doi:10.1371/journal.pgen.1004446)
Supplement: Text S1 — Supplemental methods. (DOCX) [file pgen.1004446.s012.docx]

**DNA Topoisomerase 1α Promotes Transcriptional Silencing of Transposable Elements through DNA Methylation and Histone Lysine 9 Dimethylation in *Arabidopsis***

Thanh Theresa Dinh*^,1,2,&^, Lei Gao*^,1^, Xigang Liu*^1,^^, Dongming Li^1,3^, Shengben Li^1^, Yuanyuan Zhao^1^, Michael O’Leary^1^, Brandon Le**^1^**, Robert J. Schmitz^4^, Pablo Manavella^5^, Shaofang Li^1^, Detlef Weigel^5^, Olga Pontes^6^, Joseph R. Ecker^4,7^, and Xuemei Chen^1,8,♯^

**SUPPLEMENTAL MATERIAL**

**SUPPLEMENTAL EXPERIMENTAL PROCEDURES**

**Plant Strains**

The two luciferase-based reporter lines, *LUCH* [[1](#_ENREF_1)] and *LUCL* [[2](#_ENREF_2)], were in the *rdr6-11* background, which prevents posttranscriptional gene silencing of the transgenes [[3](#_ENREF_3),[4](#_ENREF_4),[5](#_ENREF_5)]. *Pro_35S_:miR-LUC Pro_35S_: LUC*, another luciferase-based reporter line, was in the Col-0 background and was used as a reporter for miRNA function [[6](#_ENREF_6)]. *top1α*-*2* (Xigang Liu and Xuemei Chen, unpublished) was in the L*er* and *top1α*-*7* (Salk_112625) in the Col-0 background. Other strains, such as *nrpd1-3* [[7](#_ENREF_7)] and *nrpe1-11* [[8](#_ENREF_8)], were in the Col-0 background. *cmt3-7* [[9](#_ENREF_9)], *kyp-2* [[10](#_ENREF_10)], and *Myc-AGO4* [[11](#_ENREF_11)] were generated in the L*er* background.

To generate *TOP1α:TOP1a-HA top1α*-*2*, the *TOP1α* genomic region was amplified using *TOP1αgenoF*/*TOP1αgenoR* primers (see supplemental Table S8 for their sequences) and L*er* genomic DNA as the template. The PCR product was cloned into *pENTR/D-TOPO* (Invitrogen). After confirmation of the clone by sequencing, the insert was recombined into *pEarleyGate301* [[12](#_ENREF_12)] using a Gateway LR Clonase kit (Invitrogen). The *TOP1α:TOP1a-HA* plasmid was then used to transform *top1α*-*2* plants.

**Plant Growth Conditions, Chemical Screening Conditions and Luciferase Live Imaging**

*Arabidopsis thaliana* seeds were surface sterilized by either 30% bleach or fumigated in an airtight container with a solution containing 50 mL of 100% bleach and 8% HCl for six hours. After, the seeds were planted on half-strength MS-agar plates containing 1% sucrose (no sucrose was added in plates used for chemical screening to minimize fungal growth), stratified at 4°C for two days and moved into a growth chamber. Plants were grown under continuous light at 23°C. All experiments were performed with ten-day-old seedlings. Chemical screen conditions were as previously described [[2](#_ENREF_2)]. CPT was identified as a hit compound through a screen with the LACTA library of 3580 compounds (<http://cutlerlab.blogspot.com/2008/05/latca.html>). Luciferase live imaging was performed as described [[1](#_ENREF_1)].

**Luciferase Activity Assay**

*LUCH, LUCL,* and *Pro_35S_:miR-LUC Pro_35S_: LUC* seeds were grown on selective media plates. After nine days, seedlings were transferred one by one to 96-well, 2 mL plates (Fisher) containing MS media and various chemicals. Luciferin was added immediately prior to the loading of the plates into the TopCount NXT microplate luminescence counter (Perkin Elmer). During the next 48 hours, luciferase luminescence was read every 6-15 minutes for each plate and luciferase luminescence levels were calculated via the Topcount Software Pack (Perkin Elmer).

**RT-PCR and qRT-PCR**

RNA was extracted with Tri-reagent (Trizol) per manufacturer’s instructions. 10μg of total RNA was subjected to DNaseI treatment followed by reverse transcription. RT-PCR was performed on the cDNAs using primers specific for each locus of interest (Table S6). For detecting Pol V-dependent transcripts, the RT SuperScript III kit (Invitrogen) was used and the RT reaction was performed per manufacturer’s instructions with locus-specific primers. qRT-PCR for *MEA-ISR* was performed on the same cDNAs using a Biorad real-time PCR SYBR Green system (Biorad).

**Small RNA Blots**

Total RNA was isolated as described above. For miRNA detection, 5 μg of total RNA was used, whereas 30-40 μg was used for siRNA detection. Small RNA blotting and hybridization of blots was performed as described [[13](#_ENREF_13)].

**Small RNA Library Construction**

Fifty μg total RNA was resolved on a 15% denaturing polyacrylamide gel and a gel slice containing RNAs of 15 to 40 nucleotides (based on the O’ Range Ruler 10 bp RNA ladder (Thermo Scientific)) was extracted and transferred to a 1.5 ml tube. The gel piece was ground, 500μl of 0.4N NaCl (DEPC) was added to the ground mixture, and the tube containing the gel slice was shaken overnight at 4°C. Eluted RNAs were precipitated using ethanol, re-suspended in DEPC water, and used in library construction with the Illumina TruSeq-small RNA sample preparation kit per manufacturer’s instructions (Illumina). In brief, gel-purified small RNAs were ligated sequentially to 3’ and 5’ adaptors. Following reverse transcription, PCR amplification of the cDNA resulted in the small RNA library, which was gel-purified and subjected to high throughput sequencing.

**Analysis of Small RNA High Throughput Sequencing Data**

The raw reads of small RNAs were processed by PERL scripts built in house as described previously [[14](#_ENREF_14)]. Briefly, the raw reads were screened with Illumina’s quality control filter. The reads that passed the filter were separated into different bins according to their barcodes (indexes). The adaptor was trimmed for each read. Reads that match known rRNAs, tRNAs, snRNAs, and snoRNAs were removed. Reads of 20–24 nt were selected as the raw small RNA sequences. For Col-0, *nrpd1-3*, and *nrpe1-11*, which are in the Columbia ecotype, the small RNA reads were mapped to the Tair10 *Arabidopsis* genome with SOAP2 [[15](#_ENREF_15)]. For L*er* and *top1α*, which are in the Landsberg ecotype, the small RNA reads were mapped to a pseudo-L*er* genome generated by incorporating the L*er* polymorphisms into the Tair10 Columbia genome (<ftp://ftp.arabidopsis.org/Polymorphisms/Ecker_ler.homozygous_snp.txt>). This enables the direct comparison of small RNA regions between the Columbia and Landsberg samples.

**Identification of DSRs (Differential Small RNA Regions)**

In order to identify DSRs, every chromosome of *Arabidopsis* was divided into continuous 500 bp windows. Small RNAs whose 5’ nucleotide falls into a 500 bp window were counted as those belonging to this window. The number of reads in every window was recorded and served as the abundance of small RNAs in this window. The reads were normalized to RPM (reads per million). Each mutant was compared with the corresponding wild type. The windows with less than 10 RPMs in both of the libraries in a wild type/mutant pair were excluded from subsequent analysis. P-values were calculated by the Audic-Claverie method [[16](#_ENREF_16)]. The P-values were adjusted as described [[17](#_ENREF_17)] to derive the false discovery rate (FDR). Fold change > 4 and adjusted p-value (FDR) < 0.05 were required to identify DSRs between two genotypes.

**McrBC-Based DNA Methylation Assays**

Ten-day-old seedlings were harvested and DNA was isolated via CTAB extraction [[18](#_ENREF_18)]. 500ng was subjected to McrBC (New England Biolabs) treatment in the presence of GTP (New England Biolabs) for two hours and 1μl was used for subsequent qPCR reactions (for primer sequences, see Table S6). For qPCR analysis, all loci were normalized against either At2g19920 or At1g40129 as an internal, unmethylated control. At2g19920 was previously established as an unmethylated locus [[19](#_ENREF_19),[20](#_ENREF_20)]. At1g40129 was found to lack any DNA methylation in our MethylC-seq (this study).

**Southern Blotting**

5 or 10μg of CTAB-extracted DNA was subjected to *Hpa*II, *Hae*III or *Msp*I (New England Biolabs) digestion overnight at 37°C and resolved in a 1% agarose gel. Probes to detect *MEA-ISR* [[21](#_ENREF_21)], 180bp repeats [[22](#_ENREF_22)] and *5S* rDNA [[23](#_ENREF_23)] were generated as previously described. Subsequent gel processing, DNA transfer to membrane, hybridization and analyses steps were performed as previously described [[1](#_ENREF_1)].

**DNA Extraction and Bisulfite Conversion**

For analysis of DNA methylation at a single locus, 2μg of RNase-treated CTAB DNA was subjected to bisulfite conversion per manufacturer’s instructions (Qiagen). The *LUCL* locus was amplified (for primers see Table S6) with Crimson Taq (New England Biolabs) as follows: 94°C for 5 min.; 94°C for 30 sec, 56°C for 3 min, 68°C for 3 min, these steps are repeated an additional four times; 94°C for 30 sec, 56°C for 1min 30 sec, 68°C for 2 min, these steps are repeated an additional 39 times; final extension at 68°C for 5 min. The PCR product was then gel-purified (Zymo Research) and subjected to standard cloning methods using pGEM T-easy vector (Promega). Positive clones were identified and 26 clones were sequenced for each genotype. Only unique clones were processed and analyzed with <http://katahdin.mssm.edu/kismeth/revpage.pl>.

**Bisulfite Sequencing (BS-seq) Library Construction and Sequencing**

For whole-genome bisulfite sequencing, approximately 1μg of genomic DNA was sonicated to 100~300 bp with Diagenode Bioruptor using the following settings: intensity=high, on=30s, off=30s, total time=60min. Sonicated DNA was purified using the PureLink® PCR Purification Kit (Life Sciences). Purified DNA was end-repaired using the End-It kit (Epicentre) except that dCTP was not included in the reaction. The end-repaired DNA was purified with Agencourt AMPure XP beads (Beckman Coulter), A-tailed with dATP and Klenow 3’-5’ exo- (New England Biolabs) for 30 min at 37°C and then purified with Agencourt AMPure XP beads.  The purified DNA was ligated overnight at 16°C to genomic DNA adapters from the Illumina Truseq DNA sample preparation kit with T4 DNA Ligase (New England Biolabs).  Ligation products were purified with AMPure XP beads (Beckman) twice.  Less than 400ng ligated product was converted using the MethylCode Kit (Invitrogen) following the manufacturer’s guidelines except that 12ug carrier RNA (Qiagen) was added into the conversion product before column purification. The final conversion product was amplified using PfuTurbo Cx Hotstart DNA Polymerase (Agilent) under the following PCR conditions (2 minutes at 95°C, 9 cycles of 15 seconds at 98°C, 30 seconds at 60°C, 4 minutes at 72°C and 10 minutes at 72°C). The PCR product was purified with AMpure XP beads to obtain the final library DNA. BS-seq libraries were sequenced at 101 cycles using an Illumina HiSeq 2000.

**DMR Distribution Across Genes, TEs and Intergenic Regions**

The annotation of genes and TEs was according to TAIR10 (<http://www.arabidopsis.org/>). DMRs were first mapped against TEs, and the non-overlapping DMRs were further mapped against genes. The DMRs not overlapping with either TEs or genes were defined as intergenic DMRs.

**SUPPLEMENTAL REFERENCES**

1. Won SY, Li S, Zheng B, Zhao Y, Li D, et al. (2012) Development of a luciferase-based reporter of transcriptional gene silencing that enables bidirectional mutant screening in *Arabidopsis thaliana*. Silence 3: 6.

2. Dinh TT, O'Leary M, Won SY, Li S, Arroyo L, et al. (2013) Generation of a luciferase-based reporter for CHH and CG DNA methylation in *Arabidopsis thaliana*. Silence 4: 1.

3. Dalmay T, Hamilton A, Rudd S, Angell S, Baulcombe DC (2000) An RNA-dependent RNA polymerase gene in *Arabidopsis* is required for posttranscriptional gene silencing mediated by a transgene but not by a virus. Cell 101: 543-553.

4. Mourrain P, Beclin C, Elmayan T, Feuerbach F, Godon C, et al. (2000) *Arabidopsis SGS2* and *SGS3* genes are required for posttranscriptional gene silencing and natural virus resistance. Cell 101: 533-542.

5. Peragine A, Yoshikawa M, Wu G, Albrecht HL, Poethig RS (2004) *SGS3* and *SGS2*/*SDE1*/*RDR6* are required for juvenile development and the production of trans-acting siRNAs in *Arabidopsis*. Genes & Development 18: 2368-2379.

6. Manavella PA, Hagmann J, Ott F, Laubinger S, Franz M, et al. (2012) Fast-forward genetics identifies plant CPL phosphatases as regulators of miRNA processing factor HYL1. Cell 151: 859-870.

7. Herr AJ, Jensen MB, Dalmay T, Baulcombe DC (2005) RNA polymerase IV directs silencing of endogenous DNA. Science 308: 118-120.

8. Kanno T, Huettel B, Mette MF, Aufsatz W, Jaligot E, et al. (2005) Atypical RNA polymerase subunits required for RNA-directed DNA methylation. Nature Genetics 37: 761-765.

9. Lindroth AM, Cao X, Jackson JP, Zilberman D, McCallum CM, et al. (2001) Requirement of CHROMOMETHYLASE3 for maintenance of CpXpG methylation. Science 292: 2077-2080.

10. Jackson JP, Lindroth AM, Cao X, Jacobsen SE (2002) Control of CpNpG DNA methylation by the KRYPTONITE histone H3 methyltransferase. Nature 416: 556-560.

11. Li CF, Pontes O, El-Shami M, Henderson IR, Bernatavichute YV, et al. (2006) An ARGONAUTE4-containing nuclear processing center colocalized with Cajal bodies in Arabidopsis thaliana. Cell 126: 93-106.

12. Earley KW, Haag JR, Pontes O, Opper K, Juehne T, et al. (2006) Gateway-compatible vectors for plant functional genomics and proteomics. Plant J 45: 616-629.

13. Zheng B, Wang Z, Li S, Yu B, Liu JY, et al. (2009) Intergenic transcription by RNA polymerase II coordinates Pol IV and Pol V in siRNA-directed transcriptional gene silencing in *Arabidopsis*. Genes & Development 23: 2850-2860.

14. Lertpanyasampatha M, Gao, L., Kongsawaworaku, P., Viboonjun, U., Chrestin, H., Liu, R., Chen, X., Narangajavana, J. (2012) Genome-wide analysis of microRNAs in rubber tree (*Hevea brasiliensis L*.) using high-throughput sequencing. Planta.

15. Li R, Yu C, Li Y, Lam TW, Yiu SM, et al. (2009) SOAP2: an improved ultrafast tool for short read alignment. Bioinformatics 25: 1966-1967.

16. Audic S, Claverie JM (1997) The significance of digital gene expression profiles. Genome Research 7: 986-995.

17. Benjamini Y, Hochberg Y (1995) Controlling the false discovery rate: a practical and powerful approach to multiple testing. Journal of the Royal Statistical Society Series B 57: 289-300.

18. Rogers SO, Bendich AJ (1985) Extraction of DNA from milligram amounts of fresh, herbarium and mummified plant tissues. Plant Molecular Biology 5: 69-76.

19. Gao Z, Liu HL, Daxinger L, Pontes O, He X, et al. (2010) An RNA polymerase II- and AGO4-associated protein acts in RNA-directed DNA methylation. Nature 465: 106-109.

20. Hamilton A, Voinnet O, Chappell L, Baulcombe D (2002) Two classes of short interfering RNA in RNA silencing. The EMBO journal 21: 4671-4679.

21. Cao X, Jacobsen SE (2002) Locus-specific control of asymmetric and CpNpG methylation by the DRM and CMT3 methyltransferase genes. Proc Natl Acad Sci U S A 99 Suppl 4: 16491-16498.

22. Vongs A, Kakutani T, Martienssen RA, Richards EJ (1993) Arabidopsis thaliana DNA methylation mutants. Science 260: 1926-1928.

23. Lahmy S (2009) PolV(PolIVb) function in RNA-directed DNA methylation requires the conserved active site and an additional plant-specific subunit. Proc Natl Acad Sci USA 106: 941-946.

**SUPPLEMENTAL TABLES**

**Table S1. Summary of bisulfite conversion efficiency for each genotype**

|  | **CG** | **CHG** | **CHH** | **Total C** |
| --- | --- | --- | --- | --- |
| **Col-0 A*** | 98.0% | 97.9% | 97.8% | 97.8% |
| **Col-0 B*** | 98.2% | 98.1% | 97.8% | 97.9% |
| **Col-0 C*** | 98.0% | 97.9% | 97.8% | 97.9% |
| ***nrpd1-3* B** | 98.0% | 98.0% | 97.8% | 97.9% |
| ***nrpd1-3* C** | 97.9% | 97.8% | 97.7% | 97.7% |
| ***nrpe1-11* B** | 98.1% | 98.1% | 97.9% | 97.9% |
| ***nrpe1-11* C** | 98.0% | 98.0% | 97.9% | 97.9% |
| ***top1α-7* A** | 98.0% | 97.9% | 97.8% | 97.9% |
| **L*er* A** | 97.7% | 97.7% | 97.6% | 97.6% |
| ***top1α-2* A** | 97.6% | 97.6% | 97.5% | 97.5% |

* “A”, “B”, and “C” denote different biological replicates. All samples with the same letter notation were processed at the same time with the biological materials grown at the same time and in the same manner.

**Table S2. Read coverage of whole genome bisulfite sequencing libraries**

| **CHH** | **# of sequenced ^m^C** | **# of total sequenced C** | **31198380 *** |
| --- | --- | --- | --- |
|  |  |  | **Coverage^&^** |
| **Col-0 A^§^** | 12949522 | 351132278 | 11.255 |
| **Col-0 B^§^** | 6974394 | 228191510 | 7.314 |
| **Col-0 C^§^** | 8081587 | 276670598 | 8.868 |
| ***top1α-7* A** | 10469168 | 316538238 | 10.146 |
| **L*er* A** | 11536699 | 291162622 | 9.333 |
| ***top1α-2* A** | 11304607 | 298325796 | 9.562 |
| ***nrpd1-3* B** | 3996254 | 221784087 | 7.109 |
| ***nrpd1-3* C** | 3928539 | 238723704 | 7.652 |
| ***nrpe1-11* B** | 5488089 | 293648870 | 9.412 |
| ***nrpe1-11* C** | 3147078 | 175298018 | 5.619 |
|  |  |  |  |
| **CG** | **# of sequenced ^m^C** | **# of total sequenced C** | **5567714 **** |
|  |  |  | **Coverage^&^** |
| **Col-0 A** | 20359074 | 63064636 | 11.327 |
| **Col-0 B** | 11967876 | 40092653 | 7.201 |
| **Col-0 C** | 14292137 | 48943906 | 8.791 |
| ***top1α-7* A** | 17407808 | 57735189 | 10.370 |
| **L*er* A** | 16292750 | 54895136 | 9.860 |
| ***top1α-2* A** | 15579371 | 55729079 | 10.009 |
| ***nrpd1-3* B** | 11233072 | 40655236 | 7.302 |
| ***nrpd1-3* C** | 11410979 | 42322601 | 7.601 |
| ***nrpe1-11* B** | 14992909 | 51859716 | 9.314 |
| ***nrpe1-11* C** | 8591198 | 30902224 | 5.550 |
|  |  |  |  |
| **CHG** | **# of sequenced ^m^C** | **# of total sequenced C** | **6093657 ***** |
|  |  |  | **Coverage^&^** |
| **Col-0 B** | 7756135 | 66779144 | 10.959 |
| **Col-0 A** | 4368090 | 43083879 | 7.070 |
| **Col-0 C** | 5122123 | 52159761 | 8.560 |
| ***top1α-7* A** | 6866937 | 61918007 | 10.161 |
| **L*er* A** | 6504312 | 59145337 | 9.706 |
| ***top1α-2* A** | 6227381 | 60355438 | 9.905 |
| ***nrpd1-3* B** | 3536815 | 43885788 | 7.202 |
| ***nrpd1-3* C** | 3458560 | 45355541 | 7.443 |
| ***nrpe1-11* B** | 4982617 | 55895446 | 9.173 |
| ***nrpe1-11* C** | 2750976 | 33011274 | 5.417 |
|  |  |  |  |
| **Total** | **# of sequenced ^m^C** | **# of total sequenced C** | **42859751 ****** |
|  |  |  | **Coverage^&^** |
| **Col-0 B** | 41064731 | 480976058 | 11.222 |
| **Col-0 A** | 23310360 | 311368042 | 7.265 |
| **Col-0 C** | 27495847 | 377774265 | 8.814 |
| ***top1α-7* A** | 34743913 | 436191434 | 10.177 |
| **L*er* A** | 34333761 | 405203095 | 9.454 |
| ***top1α-2* A** | 33111359 | 414410313 | 9.669 |
| ***nrpd1-3* B** | 18766141 | 306325111 | 7.147 |
| ***nrpd1-3* C** | 18798078 | 326401846 | 7.616 |
| ***nrpe1-11* B** | 25463615 | 401404032 | 9.366 |
| ***nrpe1-11* C** | 14489252 | 239211516 | 5.581 |

* # of total CHH sites in genome ** # of total CG sites in genome *** # of total CHG sites in genome **** # of total C sites in genome

**^&^** Coverage = # of total sequenced C/# of total CXX sites in genome

**^§^** “A”, “B”, and “C” denote different biological replicates. All samples with the same letter notation were processed at the same time with the biological materials grown at the same time and in the same manner.

**Table S3. Correlation coefficient values for the different biological replicates of each genotype in MethylC-seq**

|  | **Col-0 A** | **Col-0 B** | **Col-0 C** |  |
| --- | --- | --- | --- | --- |
| **Col-0 A^§^** |  | 0.997 | 0.997 |  |
| **Col-0 B^§^** | 0.997 |  | 0.997 |  |
| **Col-0 C^§^** | 0.997 | 0.997 |  |  |
|  | ***nrpd1-3* C** |  |  | |
| ***nrpd1-3* B** | 0.998 |  |  | |
|  | ***nrpe1-11* C** |  |  | |
| ***nrpe1-11 B*** | 0.997 |  |  | |

**^§^** “A”, “B”, and “C” denote different biological replicates. All samples with the same letter notation were processed at the same time with the biological materials grown at the same time and in the same manner.

| **Table S4. DMRs between wild-type samples** | | | | | | |
| --- | --- | --- | --- | --- | --- | --- |
|  |  |  |  |  |  |  |
| A. Number of DMRs between any two Col replicates in this study | | | | |  |  |
|  |  | CG | CHG | CHH |  |  |
|  | Col A vs. Col B | 21 | 8 | 2139 |  |  |
|  | Col A vs. Col C | 66 | 19 | 3452 |  |  |
|  | Col B vs. Col C | 10 | 2 | 1708 |  |  |
|  |  |  |  |  |  |  |
|  |  |  |  |  |  |  |
| B. Number of DMRs between any two wild-type (WT) samples in a published study** | | | | | | |
|  |  | CG | CHG | CHH |  |  |
|  | WT1 vs. WT2 | 1978 | 2804 | 15393 |  |  |
|  | WT1 vs. WT3 | 1762 | 213 | 4151 |  |  |
|  | WT2 vs. WT3 | 446 | 550 | 15392 |  |  |
|  |  |  |  |  |  |  |
|  | ** Stroud et al. (2013). Cell 152, 1-13 | | |  |  |  |

**Table S7. Only a small number of differential small RNA regions (DSRs) were found between wild type and *top1α-2.***

Whole genome high throughput sequencing was performed for small RNAs in wild type (Col-0 and L*er*), *nrpd1-3,* a Pol IV mutant, *nrpe1-11*, a Pol V mutant, and *top1α-2.* *nrpd1-3* and *nrpe1-11* are to be compared to Col-0 and *top1α-2* is to be compared to L*er*. The genome was divided into 500bp static windows and small RNA reads in each window were counted and compared between each mutant and its corresponding wild type. Thousands of DSRs were found in *nrpd1-3* or *nrpe1-11* as compared to Col-0, but only 71 were found in *top1α-2* relative to L*er* (see Experimental Procedures for the derivation of DSRs). The numbers of DSRs mapping to different genomic features (TE, gene, and inergenic region) are listed. TE= transposable element. “Reduced” and “increased” refer to DSRs with reduced and increased small RNA read count in the mutants, respectively.

|  | ***nrpd1-3*/Col-0** | ***nrpe1-11*/Col-0** | ***top1α-2*/L*er*** |
| --- | --- | --- | --- |
| **>=10 RPM*** | 10729 | 13961 | 10066 |
| **reduced** | ***nrpd1-3*/Col-0** | ***nrpe1-11*/Col-0** | ***top1α-2*/L*er*** |
| **TE** | 5746 | 2089 | 25 |
| **gene** | 681 | 352 | 15 |
| **intergenic** | 2056 | 1083 | 31 |
| **total** | 8483 | 3524 | 71 |
|  |  |  |  |
| **increased** | ***nrpd1-3*/Col-0** | ***nrpe1-11*/Col-0** | ***top1α-2*/L*er*** |
| **TE** | 9 | 294 | 106 |
| **gene** | 27 | 31 | 20 |
| **intergenic** | 14 | 60 | 17 |
| **total** | 50 | 385 | 143 |

* The numbers of windows with at least 10 RPM (reads per million) of small RNAs in one of the two libraries. Only these windows were used in subsequent DSR derivation.

**Table S8. Oligonucleotides used in this study**

| **Name** | **Oligonucleotide Sequence** | **Application** |
| --- | --- | --- |
|  |  |  |
| **lucp6** | 5'-GCACCCGGGGAAGACGCCAAAAACATAAAAGAAA-3' | McrBC-PCR, Southern blot |
| **lucp7** | 5'-GGACCCGGGTGCGATCTTTCCGCCCTTCTTGGCCT-3' | McrBC-PCR, Southern blot |
| **Actin1-F** | 5'-CCAAGCAGCATGAAGATCAA-3' | McrBC-PCR |
| **Actin1-R** | 5'-TGAACAATCGATGGACCTGA-3' | McrBC-PCR |
| **35Sf** | 5'-CAAAGCAAGTGGATTGATGTGA-3' | McrBC-PCR, Southern blot |
| **35Sr** | 5'-TTTCCACGATGCTCCTCGT-3' | Southern blot |
| **LUC 0.13k R** | 5'-TATGTGCATCTGTAAAAGCAA-3' | McrBC-PCR |
| **YZ 35S Bis F** | 5'-AttAtTGTyGGtAGAGGtATtTTGAAyGATAGtt-3' | Bisulfite sequencing |
| **YZ LUC Bis R** | 5'-CATCTaTAAAAaCAATTaTTCCAaaAACCAaa-3' | Bisulfite sequencing |
| **N_UBQ5** | 5'-GGTGCTAAGAAGAGGAAGAAT-3' | RT-PCR, loading control |
| **C_UBQ5** | 5'-CTCCTTCTTTCTGGTAAACGT-3' | RT-PCR, loading control |
| **LUCmF5** | 5'-CTCCCCTCTCTAAGGAAGTCG-3' | RT-PCR for LUC |
| **LUCmR5** | 5'-CCAGAATGTAGCCATCCATC-3' | RT-PCR for LUC |
| **At2g19990-F** | 5'-TCACCCGAACAGTTGGAAGAA-3' | McrBC-PCR |
| **At2g19990-R** | 5'-GTGAGGAACCGGTCCATTATTGCT-3' | McrBC-PCR |
| **Cluster4-F1** | 5'-CGTCCTCAAAGTTCCAGAGAT -3' | qRT-PCR |
| **Cluster4-R1** | 5'-CGGTATTCTCCATCCCAAAG -3' | qRT-PCR |
| **AtCopia2-F1** | 5'-TTGCCCCAACAACAAAAA -3' | qRT-PCR |
| **AtCopia2-R1** | 5'-CAGAGAAAGAGATAGAAGAAATGA -3' | qRT-PCR |
| **AtMuI-F1** | 5'-GGCAGTCGGTTTGTCATTCT -3' | qRT-PCR |
| **AtMuI-R1** | 5'-CCTTCTTGGCATGGTTCTTC -3' | qRT-PCR |
| **MEA ISR- F1** | 5'-CGCGAACGACTATTGCTAAA -3' | qRT-PCR B region |
| **MEA ISR- R1** | 5'-ACGATTCCACAAATCCAACA-3' | qRT-PCR B region |
| **MEA-RT-R** | 5'-TGAAATCTAACCGGATTTTGG -3' | Gene specific primer for RT B region |
| **LNA-siR1003** | 5'-A+TGC+CAA+GTT+TGG+CCT+CAC+CGT+C-3' | Probe for northern blot |
| **LNA-cluster4** | 5'-AA+GATC+AAAC+ATCA+GCA+GCGTC+AG+AGG+CTT-3' | Probe for northern blot |
| **SoloLTR** | 5'-GGATTCACGATTAGAGAACGTAGA-3' | Probe for northern blot |
| **LNA-miR173** | 5'-GT+GAT+TTC+TCT+CTG+TAA+GCG+AA-3' | Probe for northern blot |
| **ACTIN60-F** | 5'-ATCCCTCAGCACCTTCCAAC-3' | qRT-PCR; control |
| **ACTIN60-R** | 5'-AAAATCCACATAACAACAGATAGTTCA-3' | qRT-PCR; control |
| **Chloro_up_bis F** | 5'-TATGGTGAGYTACAATAATGGTTAAAGAG-3' | Bisulfite sequencing |
| **Chloro_up_bis R** | 5'-TATCTTTACCRATTAACCAATTTCTAAAC-3' | Bisulfite sequencing |
| **IGN24-F** | 5'-CGCATACGATGGTCGGAGAGTT -3' | RT-PCR B region |
| **IGN24-R** | 5'-GCTTATCATTATCCAAACTTGATCCTATCCTAAA -3' | RT-PCR B region |
| **AtSN1-F** | 5’-CCAGAAATTCATCTTCTTTGGAAAAG-3’ | RT-PCR B region |
| **AtSN1-R** | 5’-GCCCAGTGGTAAATCTCTCAGATAGA-3’ | RT-PCR B region |
| **IGN26-F** | 5’-CTCTTTCAGTGCGACAGCCTCAT-3’ | RT-PCR B region |
| **IGN26-R** | 5’-CGGCCAGGAAACCCTAACTTCC-3’ | RT-PCR B region |
| **IGN5-F** | 5’-CGCAGCGGAATTGACATCCTATC-3’ | RT-PCR B region |
| **IGN5-R** | 5’-TCGGAAAGAGACTCTCCGCTAGAAA-3’ | RT-PCR B region |
| **IGN25-F** | 5’-CTTCTTATCGTGTTACATTGAGAACTCTTTCC-3’ | RT-PCR B region |
| **IGN25-R** | 5’-ATTCGTGTGGGCTTGGCCTCTT-3’ | RT-PCR B region |
| **IGN15-F** | 5’-CCATAGCATAGAAACTTGGCGATATATGAA-3’ | RT-PCR B region |
| **IGN15-R** | 5’-CGGAAAAGGTAAGGTGGTTGGAAAA-3’ | RT-PCR B region |
| **IGN23-F** | 5’-ACTGAAAATTGTAAACAAAGAAACGGCACTACA-3’ | RT-PCR B region |
| **IGN23-R** | 5’-GATCGGTCCATAAACTTGTTGGGTTT-3’ | RT-PCR B region |
| **AtSN1-F** | 5’-ACCAACGTGCTGTTGGCCCAGTGGTAAATC-3’ | qRT-PCR A region; ChIP-qPCR; McrBC- qPCR |
| **AtSN1-R** | 5’-AAAATAAGTGGTGGTTGTACAAGC-3’ | qRT-PCR A region; ChIP-qPCR; McrBC- qPCR |
| **AtMu1-F1** | 5’-CCGAGAACTGGTTGTGGTTT-3’ | qRT-PCR A region; ChIP-qPCR; McrBC- qPCR |
| **AtMu1-R1** | 5’-GCTCTTGCTTTGGTGATGGT-3’ | qRT-PCR A region; ChIP-qPCR; McrBC- qPCR |
| **IGN5-F** | 5’-AAGCCCAAACCATACACTAATAATCTAAT-3’ | ChIP-qPCR |
| **IGN5-R** | 5’-CCGAATAACAGCAAGTCCTTTTAATA-3’ | ChIP-qPCR |
| **IR-71 F1** | 5'-TATCATCCTTCTGGTTTTGG-3' | qRT-PCR A region; McrBC-qPCR |
| **IR-71 R1** | 5'-AAGCAACATTCATTTCAGC-3' | qRT-PCR A region; McrBC-qPCR |
| **siR02-F2** | 5'-CAATATGTTCTTCACCATCG-3' | qRT-PCR A region; McrBC-qPCR |
| **siR02-R2** | 5'-ATTTGCGAAACTAATGGAAG-3' | qRT-PCR A region; McrBC-qPCR |
| **cluster4-F** | 5'-CGTCCTCAAAGTTCCAGA-3’ | qRT-PCR A region; McrBC-qPCR |
| **cluster4-R** | 5'-GGTATTCTCCATCCCAAAG-3’ | qRT-PCR A region; McrBC-qPCR |
| **SoloLTR;IG/LINE-F** | 5’-AACTAACGTCATTACATACACATCTTG-3’ | ChIP-qPCR |
| **SoloLTR;IG/LINE-R** | 5’-AATTAGGATCTTGTTTGCCAGCTA-3’ | ChIP-qPCR |
| **AtGP1-F** | 5'-TGGTTTTTCCTGTCCAGTTTG-3’ | qRT-PCR A region; ChIP-qPCR; McrBC-qPCR |
| **AtGP1-R** | 5'-AACAATCCTAACCGGGTTCC-3’ | qRT-PCR A region; ChIP-qPCR; McrBC-qPCR |
| **EIF4A1-F** | 5'-TCTTGGTGAAGCGTGATGAG-3’ | ChIP-qPCR |
| **EIF4A1-R** | 5'-GCTGAGTTGGGAGATCGAAG-3’ | ChIP-qPCR |
| **chr2_1882324_CHH-F** | 5'-CGAGCCAAAAATTGTTGAAAT-3' | McrBC-qPCR |
| **chr2_1882324_CHH-R** | 5'-TTTGAGGGGATAGAGTATCTCTTG-3' | McrBC-qPCR |
| **TOP1αgeno-F** | 5'-CACCGGCAAGACGGATCTGGAGGG-3' | Generation of *TOP1α-HA* |
| **TOP1αgeno-R** | 5'-GGGTAAACGAGATCGTTGAATGAC-3' | Generation of *TOP1α-HA* |
